# Folic acid-modified ROS-responsive nanoparticles encapsulating luteolin for targeted breast cancer treatment

**DOI:** 10.1080/10717544.2021.1963351

**Published:** 2021-08-17

**Authors:** Yu Wang, Qianmei Wang, Wei Feng, Qian Yuan, Xiaowei Qi, Sheng Chen, Pu Yao, Qing Dai, Peiyuan Xia, Dinglin Zhang, Fengjun Sun

**Affiliations:** a Department of Pharmacy, Southwest Hospital, Army Medical University (Third Military Medical University), Chongqing, China; b Department of Chemistry, College of Basic Medicine, Army Medical University (Third Military Medical University), Chongqing, China; c Department of Breast and Thyroid Surgery, Southwest Hospital, Army Medical University (Third Military Medical University), Chongqing, China; d Department of Pediatrics, Southwest Hospital, Army Medical University (Third Military Medical University), Chongqing, China; e Department of Urology, Southwest Hospital, Third Military Medical University (Amy Medical University), Chongqing, China

**Keywords:** Luteolin, reactive oxygen species, nanoparticles, breast cancer, targeting therapy

## Abstract

Luteolin (Lut) is a natural flavonoid polyphenolic compound with multiple pharmacological activities, such as anti-oxidant, anti-inflammatory, and anti-tumor effects. However, the poor aqueous solubility and low bioactivity of Lut restrict its clinical translation. Herein, we developed a reactive oxygen species (ROS)-responsive nanoplatforms to improve the bioactivity of Lut. Folic acid (FA) was employed to decorate the nanoparticles (NPs) to enhance its targeting ability. The size of Lut-loaded ROS-responsive nanoparticles (Lut/Oxi-αCD NPs) and FA-modified Lut/Oxi-αCD NPs (Lut/FA-Oxi-αCD NPs) is 210.5 ± 6.1 and 196.7 ± 1.8 nm, respectively. Both Lut/Oxi-αCD NPs and Lut/FA-Oxi-αCD NPs have high drug loading (14.83 ± 3.50 and 16.37 ± 1.47%, respectively). *In vitro* cellular assays verified that these NPs could be efficiently internalized by 4T1 cells and the released Lut from NPs could inhibit tumor cells proliferation significantly. Animal experiments demonstrated that Lut/Oxi-αCD NPs, especially Lut/FA-Oxi-αCD NPs obviously accumulated at tumor sites, and inhibited tumor growth ∼3 times compared to the Lut group. In conclusion, the antitumor efficacy of Lut was dramatically improved by targeting delivery with the ROS-responsive nanoplatforms.

## 1. Introduction

Luteolin (Lut, 3′,4′,5,7-tetrahydoxyflavone), a natural flavonoid polyphenolic compound, is found in many fruits, vegetables, and medicinal herbs, has multiple biological activities, such as anti-microbial, anti-inflammatory, anti-allergy, anti-oxidant, and anti-cancer effects (Aziz et al., 2018). In particular, Lut shows multiple anti-tumor effects against lung, prostate, breast, glioblastoma, colon, liver, cervical, and pancreatic cancers (Wu et al., 2018; Imran et al., 2019). It can block cancer development by inhibition of tumor cell proliferation (Sato et al., 2015), activation of cell cycle arrest (Cook et al., 2016), anti-angiogenesis (Cook et al., 2015), anti-metastasis (Cook et al., 2017), and inducing apoptosis of tumor cell through a various signaling pathway. Importantly, recent studies suggested that Lut can serve as a chemopreventive and chemotherapeutic agent in breast cancer (Ahmed et al., 2019). Lut can decrease breast cancer incidence by inhibiting the formation of lipid peroxide, increasing the activities of superoxide dismutase, catalase, and glutathione peroxidase in the breast tissues (Samy et al., 2006). Alternatively, Lut can suppress pro-intravasative trigger factors, such as matrix metalloproteinase (MMP) in MDA-MB-231 breast cancer cells (Hong et al., 2018). Although Lut exhibits fascinating prospects as chemopreventive and chemotherapeutic agents for the treatment of various cancers, the clinical application of Lut is limited by its intrinsic poor aqueous solubility and the low oral bioavailability (Huang et al., 2017). Targeted delivery of Lut by nano-drug delivery systems (NDDS) may as an optional strategy to overcome the abovementioned limitations.

In the past decades, NDDS has been extensively explored due to their multiple benefits, such as improving pharmaceutical properties, reducing dosing frequency, and enhancing targeting capability (Zhang & Zhang, 2020). Some phytopharmaceuticals, such as epigallocatechin gallate (Yang et al., 2020), curcumin (Chen et al., 2020), and resveratrol (Arora & Jaglan, 2018) have been successfully fabricated into nanoformulations to optimize their physicochemical properties and enhance their efficacies. Similarly, Lut has been encapsulated by nanoparticles (NPs) (Majumdar et al., 2014), micelles (Qing et al., 2017), liposomes (Huang et al., 2017; Wu et al., 2018), and nanoemulsions (Shin et al., 2018) to improve its bioavailability and efficacy. However, Lut cannot be smartly released from these nanoformulations and the biological efficacies of Lut-loaded nanoformulations have not been systematically evaluated. In addition, overexpression of folate receptors (FRs) has been detected on activated macrophages and some cancer cells (i.e. breast, lung, kidney, and ovarian cancer cells) than on healthy cells (Fernandez et al., 2018). Folic acid (FA) shows a strong affinity to FRs, which provides an opportunity to target deliver cargos to inflammatory or cancerous tissues by FA-modified nanoformulations (Tagde et al., 2020). Alternatively, ROS level in tumor tissues has been detected thousand times higher than that of in normal tissues (Dickinson & Chang, 2011; Xu et al., 2017). Based on the abnormal microenvironment of tumor tissues, ROS-responsive nanoformulations have been broadly developed to deliver therapeutics to tumor tissues, and these payloads can be smartly released under the ROS microenvironment (Wang et al., 2019; Yin et al., 2019). Our previous works employed 4-(hydroxymethyl) phenylboronic acid pinacol ester (HPAP, a ROS responsive moiety) to modify cyclodextrin (CD) to prepare ROS-responsive biomaterials (Oxi-CD) (Zhang et al., 2015). These materials have distinguished ROS-responsiveness and favorable biocompatibility, which have been successfully fabricated nanoformulations to encapsulate moxifloxacin and dexamethasone to target the treatment of pulmonary infection and rheumatoid arthritis, respectively (Wang et al., 2019; Ni et al., 2020).

**Figure 1.**
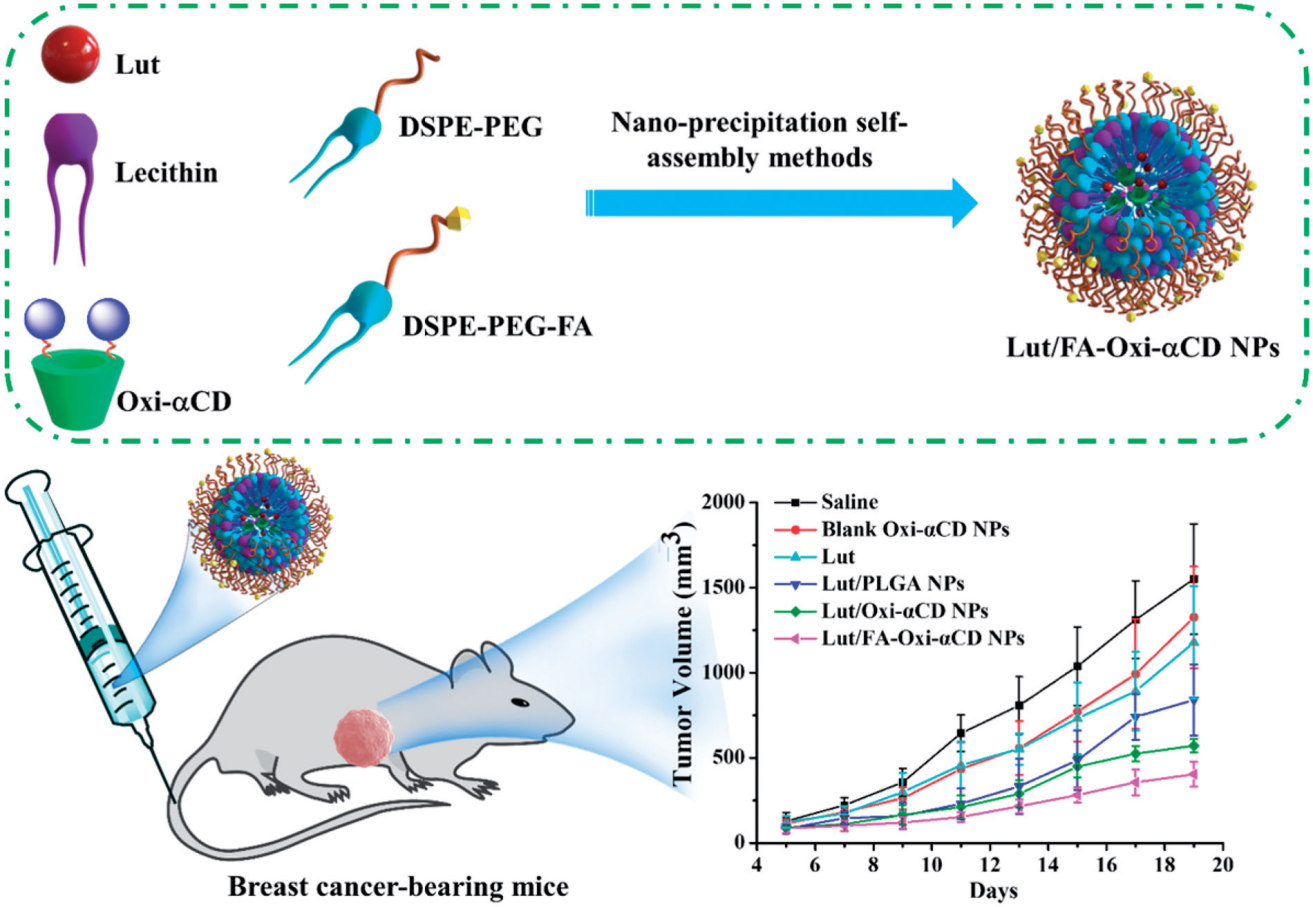
Fabrication of Lut-loaded ROS-responsive NPs and their *in vivo* anti-tumor application in a 4T1 tumor-bearing mouse model.

**Scheme 1.**
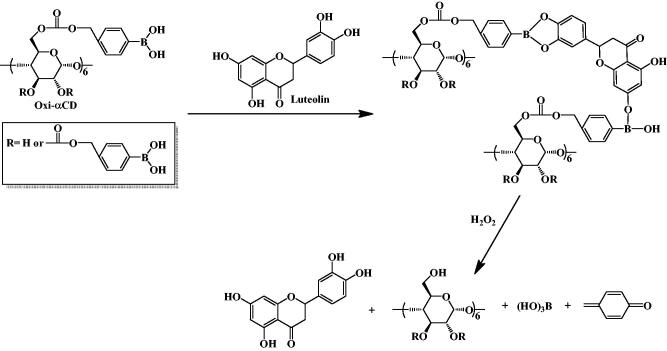
Lut forms a boronic ester bond with Oxi-αCD, and Lut is released under H_2_O_2_.

In addition, boronate exhibits a strong affinity with compounds containing 1,2-diols, such as pinacol, carbohydrate, and polyphenol. For example, Lut owing several phenolic hydroxyls which appear intensive affinity with boronate. Using boronate to modify carriers to load 1,2-diols-containing therapeutics may obtain satisfactory drug loading. To improve the bioactivity of Lut, we develop nanoplatforms for targeted delivery of Lut to pathological sites. We hypothesize that Lut has an intense affinity to our prepared ROS-responsive materials (Oxi-αCD), which can improve the delivery performance of Lut by using this material as a carrier (Scheme 1). To verify this hypothesis, we employed Oxi-αCD as a carrier to encapsulate Lut to fabricate core-shell NPs *via* a nanoprecipitation/self-assembly method (Figure 1). The periphery of the NPs was decorated with DSPE-PEG and DSPE-PEG-FA to achieve breast cancer cell targeting and long circulation in the body. The biodistribution of Lut-loaded ROS-responsive NPs was evaluated on 4T1 tumor-bearing mouse models by using *in vivo* imaging system. Anti-tumor activities of Lut-loaded NPs were evaluated both *in vitro* and *in vivo*. Our results demonstrated that Lut-loaded ROS-responsive NPs with satisfying drug loading have been successfully fabricated, and the loaded Lut can be smartly released from NPs at the pathological concentration of ROS. *In vitro* cellular assays demonstrated that Lut-loaded NPs could be effectively internalized by 4T1 tumor cells. *In vivo* image results indicated that Lut/Oxi-αCD NPs, especially Lut/FA-Oxi-αCD NPs observably accumulated at tumor sites. Both *in vitro* and *in vivo* experiments indicated that Lut/FA-Oxi-αCD NPs had enhanced anti-tumor activities compared to free Lut and non-targeted Lut/Oxi-αCD NPs.

## 2. Materials and methods

### 2.1. Materials and reagents

Pluronic F127 and poly (lactic-co-glycolic acid) (PLGA) (50:50, MW: 24,000–38,000 Da) were purchased from Sigma-Aldrich Co. (St. Louis, MO, USA). Lecithin (refined) was supplied by Alfa Aesar (Ward Hill, MA, USA). 1,2-Distearoyl-sn-glycero-3-phosphoethanolamine-N-methoxy(polyethylene glycol)-2000 (DSPE-PEG_2000_), folic acid-conjugated 1,2-distearoyl-sn-glycero-3-phosphoethanolamine-N-methoxy(polyethylene glycol)-3400 (DSPE-PEG_3400_-FA) and polystyrene microsphere were obtained from Xi’an Ruixi Corporation (Xi’an, China). Cy5-NHS ester and Cy5 free acid were purchased from Lumiprobe, LLC. (Hallandale Beach, FL, USA). Lut was obtained from Meilun Biotechnology Co. (Dalian, China). Fetal bovine serum (FBS) and Dulbecco’s modified Eagle’s medium (DMEM) were provided by HyClone Inc. (Waltham, MA, USA). 4′,6-Diamidino-2-phenylindole (DAPI), and Hydrogen Peroxide Assay Kit (S0038) were purchased from Beyotime Biotechnology Co., Ltd. (Shanghai, China). Streptomycin-penicillin solution was supplied by Solarbio Life Sciences Co., Ltd. (Beijing, China). ROS-responsive materials (Oxi-αCD) and Cy5-labeled Oxi-αCD were prepared and characterized as previously reported (Wang et al., 2019; Ni et al., 2020). The synthesis methods were also listed in the Supporting Information.

### 2.2. Fabrication and characterization of Lut-encapsulated NPs

A modified nanoprecipitation/self-assembly method was utilized to fabricate Lut-loaded NPs (Chan et al., 2009). In brief, lecithin (4.0 mg) and DSPE-PEG_2000_ (6.0 mg) were dispersed in anhydrous ethanol (400 µL) and deionized water (10.0 mL) and then heated to 65 °C for 30 min under gentle stirring (100 rpm). In parallel, Lut (5.0 mg) and Oxi-αCD (50.0 mg) were dissolved in methanol (1.0 mL) and dimethyl sulfoxide (DMSO) (1.0 mL). The drug-containing solution was dropped into the above-preheated lipid dispersion solution (1.0 mL/min) followed by vortexing for 3 min (800 rpm). After self-assembly with stir slowly for 2 h at room temperature, Lut/Oxi-αCD NPs were collected by centrifugation at 15000 rpm for 10 min. The harvested NPs were washed with 5% F127 (10.0 mL) and resuspended in 0.2 mL ultrapure water. Cy5-labeled Oxi-αCD NPs (Cy5-labeled Oxi-αCD as a carrier) and blank Oxi-αCD NPs (without Lut-encapsulated) were prepared following similar procedures (Supporting Information). In addition, FA-modified Lut/Oxi-αCD NPs (Lut/FA-Oxi-αCD NPs) were prepared with a similar method, except that DSPE-PEG_2000_ (4.0 mg) and DSPE-PEG_3400_-FA (4.0 mg) were used to fabricate the NPs. Alternatively, an emulsion solvent evaporation method was employed to fabricate Lut/PLGA NPs (Supporting Information). The size, polydispersity index (PDI), and zeta potential of NPs were determined by dynamic light scattering (DLS) and laser Doppler anemometry, using a Malvern Zetasizer (Nano ZS, Malvern, UK). The morphology of NPs was imaged by transmission electron microscopy (TEM) (JEM-1400, Japan). The FT-IR spectra of Lut, blank NPs, Lut/Oxi-αCD NPs, and Lut/FA-Oxi-αCD NPs were recorded on a PerkineElmer FT-IR spectrometer (100S). To check the stability of NPs, the fresh prepared NPs were diluted into ultrapure water or 10% FBS, following the size and PDI distribution were detected at interval time points using a Malvern Zetasizer.

### 2.3. Drug loading, encapsulation efficiency, and drug release behavior *in vitro*

To quantify the amount of Lut encapsulated in Oxi-αCD NPs, 20 μL of fresh NPs suspension was lyophilized, weighed, and dissolved in 0.2 mL of DMSO and 0.8 mL of methanol. The concentration of Lut was measured by high-performance liquid chromatography (HPLC). Drug loading (DL) and encapsulation efficiency (EE) of Lut in Oxi-αCD NPs were calculated according to the following equations:

$$\begin{array}{c} \text{DL}\%=\left(\text{Amount}\text{of}\text{Lut}\text{in}\text{NPs}/\text{Weight}\text{of}\text{NPs}\right)\times 100\%\\ \text{EE}\%=\left(\text{Amount}\text{of}\text{Lut}\text{in}\text{NPs}/\text{Weight}\text{of}\text{feeding}\text{Lut}\right)\times 100\% \end{array}$$

To investigate the Lut release profiles of NPs *in vitro*, 150 µL of newly prepared Lut/Oxi-αCD NPs or Lut/FA-Oxi-αCD NPs was transferred into dialysis tubing (MWCO: 3500 Da), which was immersed into 40.0 mL of PBS with or without 0.5 mM H_2_O_2_ and incubated at 37 °C on a platform shaker (100 rpm). At predetermined time points, 4.0 mL of supernatant was collected and replaced with 4.0 mL of fresh medium. The concentration of Lut was analyzed by HPLC and the cumulative drug release percentage was calculated.

### 2.4. Cytotoxicity evaluation by CCK-8 assay

Mouse breast cancer cell line 4T1 was obtained from the Cell Bank of the Chinese Academy of Sciences (Shanghai, China). 4T1 cells were incubated in DMEM cell culture supplemented with 10% (v/v) FBS, streptomycin (100 µg/mL), and 100 IU penicillin at 1 × 10^4^ cells/well in 96-well plates at 37 °C in a humidified atmosphere containing 5% CO_2_ for 24 h. To evaluate the cytotoxicity of Lut, blank NPs, and nanoformulations, 4T1 cells were co-incubated with Lut or its nanoformulations at various concentrations (5, 10, 20, 40, 60, 80, or 100 μM) for 48 h. Otherwise, cells were incubated with equal blank NPs to investigate the cytotoxicity of blank NPs. After incubation, the culture supernatant was discarded, and 110 µL of CCK-8 culture solution was added to each well and cultured for another 0.5 h. Subsequently, the absorbance of the cultures was measured at 450 nm using a Thermo Multiskan Spectrum spectrophotometer (Thermo Scientific Inc. MA, USA).

### 2.5. Hemolysis assay

The hemolysis of different nanoformulations was examined by incubation with freshly isolated sheep blood cells. Briefly, the fresh sheep blood (5.0 mL) was centrifuged at 3000 rpm for 5 min, then washed three times with PBS, and finally diluted as an erythrocyte stock solution (3%). Subsequently, 50 μL of the cell suspension was placed in each well of a 96-well plate and treated with Lut, Lut/Oxi-αCD NPs or Lut/FA-Oxi-αCD NPs for 1 h at 37 °C in 5% CO_2_ using a double dilution method starting at a concentration of 256 μg/mL. PBS and 1% Triton X-100 solution (w/v) were used as a negative and positive control, respectively. Finally, the suspension was centrifuged at 3000 rpm for 5 min, and the absorbance of the supernatant was measured at 450 nm. The results are expressed as a percentage of hemoglobin released relative to the positive control (Triton X-100). Experiments were performed in triplicate, and the results are an average of experiments. The hemolytic percentage (hemolysis %) was calculated as the following equation:

$$\text{Hemolysis}\%=\left(A_{450}\left(\text{NPs}\right)-A_{450}\left(\text{PBS}\right)\right)/\left(A_{450}\left(1\%\text{Triton}\mathrm{X-}100\right)-A_{450}\left(\text{PBS}\right)\right)\times 100\%.$$

### 2.6. Cell uptake

4T1 cells were seeded at 2 × 10^5^ cells/well in 12-well plates (with glass coverslips on the bottom) and allowed to grow for 24 h. Cells were incubated with free Cy5, Cy5/Oxi-αCD NPs or Cy5/FA-Oxi-αCD NPs for 2 and 4 h (the final concentration of Cy5 is 1 µg/mL^−1^). Alternatively, cells were incubated with a fresh culture medium as the control group. After incubation, cells were washed with cold PBS three times and fixed in 4% paraformaldehyde for 20 min. Subsequently, cell nuclei were stained with DAPI for 10 min and washed with PBS three times. Cell uptake efficiencies of Cy5 and Cy5-labeled NPs were detected by confocal laser scanning microscopy (CLSM) (Carl ZEISS LSM 780, Germany).

To verify the affinity of FA-modified NPs with 4T1 cells, 4T1 cells were incubated with FRα antibody (1:500, DF4058, Affinity Biosciences, OH, USA) for 2 h after being cultured in 24-well plates (with glass coverslips on the bottom) for 24 h. Then cells were treated with free Cy5, Cy5/Oxi-αCD NPs, or Cy5/FA-Oxi-αCD NPs for 4 h. After incubation, cells were washed, fixed, and stained as previously described. Finally, cell uptake efficiencies of Cy5 or Cy5-labeled NPs were acquired by CLSM.

### 2.7. Three-dimensional (3 D) on-top culture and penetration in tumor spheroids

The diffusion/penetration profiles of Cy5/Oxi-αCD NPs and Cy5/FA-Oxi-αCD NPs were examined in an *in vitro* three-dimensional (3 D) tumor spheroid model of 4T1 cells. Briefly, 2 × 10^5^ 4T1 cells were seeded on a confocal cuvette precoated a thin layer of Matrigel. After inoculation for 24 h, free Cy5, Cy5/Oxi-αCD NPs or Cy5/FA-Oxi-αCD NPs (1 μg/mL^−1^) were added into the wells for another 2 or 4 h incubation, in parallel, culture medium without FBS was used as control. At the determined time points, the medium was removed and the treated cells were washed with cold PBS three times, then fixed in 4% paraformaldehyde for 20 min, and rinsed with PBS three times. Subsequently, the cell nuclei were stained with DAPI for 10 min. After being washed with PBS three times, the distribution of NPs in 3 D tumor spheroids was observed by CLSM with layer scanning mode.

### 2.8. Determination of intracellular and extracellular H_2_O_2_ concentrations

To detect the intracellular and extracellular H_2_O_2_ concentrations, 4T1 cells (2 × 10^5^ cells/well) were seeded in 12-well plates and cultured for 24 h. Next, the cells were incubated with polystyrene NPs (PS NPs, non-ROS-responsiveness), Lut, blank Oxi-αCD NPs, Lut/Oxi-αCD NPs or Lut/FA-Oxi-αCD NPs (about 25 μM of Lut) for 24 h. Finally, the intracellular and extracellular concentrations of H_2_O_2_ were determined using a Hydrogen Peroxide Assay Kit according to the manufacturer’s protocol.

### 2.9. Initial evaluation of biological safety of Lut and Lut-loaded Oxi-αCD NPs

All animal experiments were performed by the Animal Management Rules of the Ministry of Health of the People's Republic of China (No. 55, 2001) and the guidelines for the Care and Use of Laboratory Animals of the Army Medical University (Chongqing, China). Female Kunming mice (about 30 g) were randomly divided into eight groups (*n* = 6). In the control group, mice received 0.1 mL of saline solution. In the Lut group, mice were injected with 10 mg/kg of Lut solution *via* the tail vein. In parallel, Lut/Oxi-αCD NPs or Lut/FA-Oxi-αCD NPs in ultrapure water was administered *via* vein injection at doses of 5, 10, and 20 mg/kg of Lut. Post-administration, mice were weighed at one interval day and their behaviors were monitored for any signs of illness each day. After two weeks, animals were sacrificed by cervical dislocation after anesthesia. Blood samples were collected for hematological analysis (Sysmex KX-21, Sysmex Co., Japan). Major organs including the heart, liver, spleen, lung, and kidneys were harvested and weighed. In addition, histopathological sections of the collected organs were made and stained with hematoxylin-eosin (H&E).

### 2.10. Establishment of a murine model of breast cancer and anti-tumor efficacy *in vivo*

Six-week-old female BALB/c mice weighing about 20 g were provided from the experimental animal center of Army Medical University (Chongqing, China) and kept in an SPF-level sterile animal room. 5 × 10^5^ 4T1 cells resuspended in sterile PBS (100 μL) were implanted into the fourth mammary fat pad (right) of mice to establish a breast tumor model. Primary tumor volumes were determined and calculated using the following formula:

$$V(mm^{3})=1/2(L\times W^{2}),$$

*L* (mm) is the longest diameter and *W* (mm) is the shortest diameter perpendicular to the length of the primary tumor. When the tumor volume grew to about 100 mm^3^, tumor-bearing mice were randomly divided into six treatment groups (*n* = 5) and intravenously injected with 0.9% saline, Lut, Lut/PLGA NPs, Lut/Oxi-αCD NPs, or Lut/FA-Oxi-αCD NPs at 10 mg/kg of Lut dose every 4 days for four consecutive times. Blank Oxi-αCD NPs groups received equal amounts of NPs. The body weights and tumor volumes were measured every 2 days. After the accomplishment of the therapy, mice were sacrificed, and the excised tumors and major organs were immersed in 4% paraformaldehyde for histological examination and immunohistochemistry analysis.

### 2.11. Biodistribution assay

A suspension of 4T1 cells (0.1 mL, 1 × 10^6^ cells) was injected into the fourth mammary fat pad (right) of 6-week-old female BALB/c mice to induce the growth of breast tumors. The *in vivo* biodistribution experiments were performed ∼2 weeks after tumor cell inoculation. When the tumor volume grew to about 300 mm^3^, Cy5, Cy5-labeled Lut/Oxi-αCD NPs, or Cy5-labeled Lut/FA-Oxi-αCD NPs was injected *via* tail vein at a Cy5 dose of 1 mg/kg. In parallel, the healthy mice were received the same volume of saline as the control. At 2, 4, 6, 8, 24, and 48 h after injection, mice were anesthetized and fluorescence images were acquired using an IVIS Spectrum living imaging system with a 640 nm excitation filter and a 700 nm emission filter (Perkin Elmer, USA). At appropriate intervals, mice were sacrificed and their tumors and major organs were gathered for *ex-vivo* imaging. A semi-quantitative analysis of fluorescence intensity within the major organs and tumor tissues was performed to obtain insight into the distribution behaviors of various NPs formulations *in vivo*. The fluorescence intensity was analyzed by the Living Imaging software (Perkin Elmer, USA).

In addition, the excised tumor tissues with Cy5, Cy5-labeled Lut/Oxi-αCD NPs, or Cy5-labeled Lut/FA-Oxi-αCD NPs treatment were collected for frozen sections. Briefly, the sections of tumor tissues were fixed with 4% paraformaldehyde for 20 min and rinsed with PBS three times. Next, the cell nuclei were stained with DAPI for 10 min, after being rinsed with PBS three times, the sections were observed by the CLSM to detect the accumulation of NPs in tumor tissues.

### 2.12. Statistical analysis

Results are expressed as the mean ± standard deviation (SD). All measurements include at least three independent experiments. Data were analyzed using one-way variance (ANOVA) with Tukey's multiple comparison test for more than three groups and Student’s *t*-test for two groups. Statistical significance was defined as *p < .05, **p < .01, and ***p < .01.

## 3. Results and discussion

### 3.1. Fabrication and characterization of NPs

Previously, we prepared HPAP-conjugated cyclodextrin (Oxi-αCD) which possessed good biocompatibility and favorable ROS-responsive ability under pathological levels of ROS (Zhang et al., 2015). The ROS-responsive materials have been used as a carrier to encapsulate rapamycin, moxifloxacin, and dexamethasone to target treatment atherosclerosis, pulmonary infection, and rheumatoid arthritis, respectively (Dou et al., 2017; Wang et al., 2019; Ni et al., 2020). Otherwise, Pan *et al.* recently reported that Lut exhibited a strong affinity with boronate (Bai et al., 2018). To target deliver Lut to diseased sites and improve its bioactivity, herein, we employed Oxi-αCD as a carrier to fabricate Lut-loaded NPs based on the strong affinity of Lut and boronate. Alternatively, FRs have been detected on the surface of breast cancer cells (i.e. 4T1 and MDA-MB-231 cells), which showed a strong affinity to FA (Choudhury et al., 2020). Therefore, FA was employed to modify nanoformulations to achieve active targeting to cancer cells. The ROS-responsive materials Oxi-αCD and Cy5-labeled Oxi-αCD were synthesized and characterized as our previously reported method (Wang et al., 2019; Ni et al., 2020) (Supporting Information, Schemes S1, S2). Using Oxi-αCD as a carrier, a modified nanoprecipitation/self-assembly method was employed to prepare Lut-loaded ROS-responsive NPs (Lut/Oxi-αCD NPs and Lut/FA-Oxi-αCD NPs) (Ni et al., 2020).

The physicochemical characterizations of blank Oxi-αCD NPs, blank FA-Oxi-αCD NPs, Lut/Oxi-αCD NPs, and Lut/FA-Oxi-αCD NPs were summarized in Table 1. DLS showed that the average size of blank Oxi-αCD NPs, blank FA-Oxi-αCD NPs, Lut/Oxi-αCD NPs, and Lut/FA-Oxi-αCD NPs were 149.8 ± 1.9, 163.2 ± 1.5, 210.5 ± 6.1, and 196.7 ± 1.8 nm, respectively (Table 1). The size of the Lut/Oxi-αCD NPs and Lut/FA-Oxi-αCD NPs have increased by about 60 and 30 nm compared to their blank counterparts, respectively. These results indicated that encapsulation of Lut could slightly increase the size of NPs. Interestingly, the statistical results showed that there was no significant difference between non-modified NPs and FA-modified NPs. The reason might be that only 6% (wt%) of DSPE-PEG-FA in total weight was used to prepare targeted NPs, which might not alter the size of NPs obviously.

**Table 1.** Physicochemical properties of various nanoformulations.

Nanoformulations	Size (nm)	PDI	Zeta potential (mv)	Drug loading (w/w %)	Encapsulation efficiency (%)
Blank Oxi-αCD NPs	149.8 ± 1.9	0.08 ± 0.02	−29.8 ± 1.8	/	/
Blank FA-Oxi-αCD NPs	163.2 ± 1.5	0.17 ± 0.03	−29.9 ± 2.9	/	/
Lut/Oxi-αCD NPs	210.5 ± 6.1	0.16 ± 0.03	−38.9 ± 0.4	14.83 ± 3.50	24.22 ± 2.41
Lut/FA-Oxi-αCD NPs	196.7 ± 1.8	0.13 ± 0.02	−41.0 ± 0.01	16.37 ± 1.47	33.67 ± 2.43
Lut/PLGA NPs	443.5 ± 8.8	0.22 ± 0.01	0.10 ± 0.09	10.46 ± 2.05	41.84 ± 8.20

**Figure 2.**
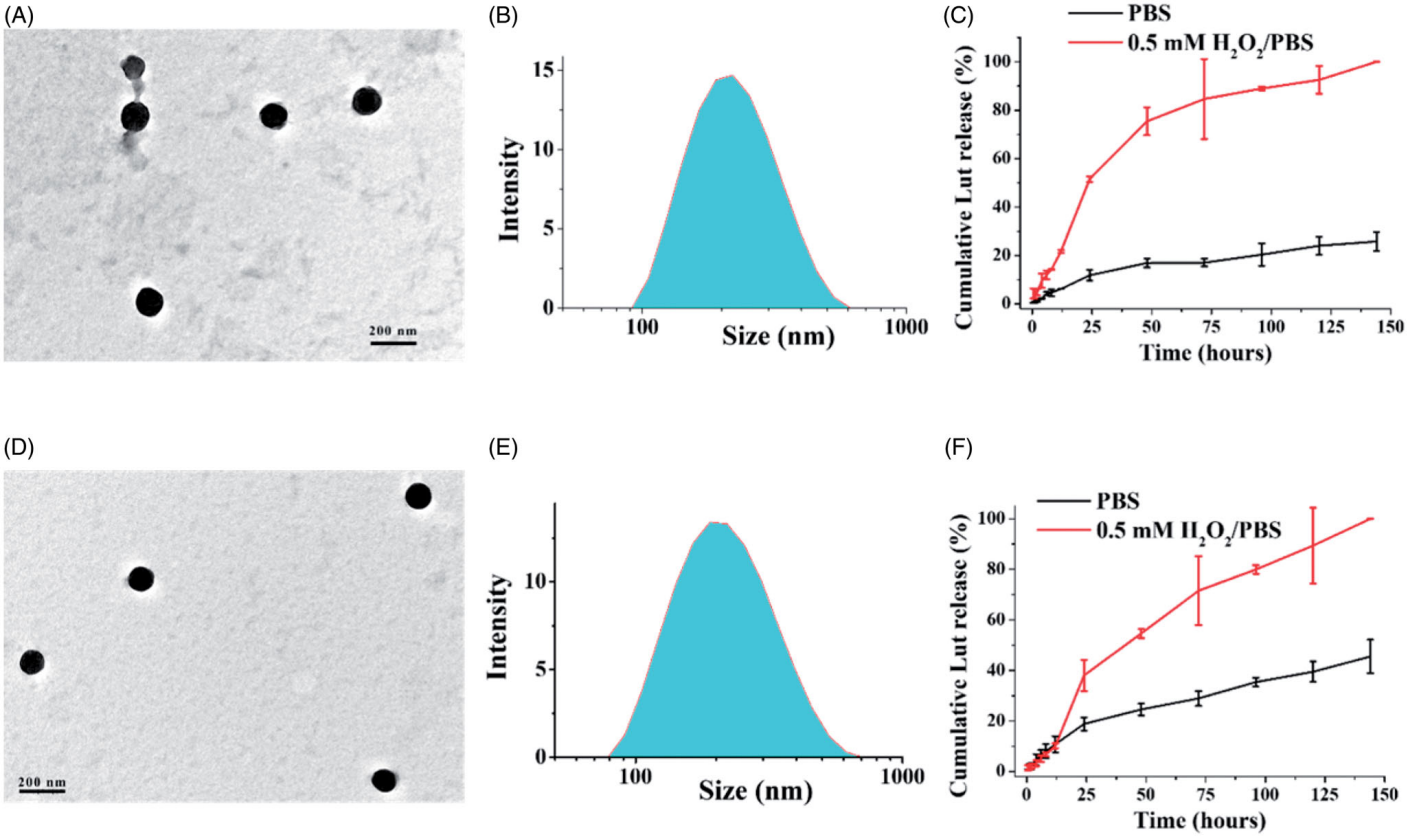
Characterization of Lut/Oxi-αCD NPs (A–C) and Lut/FA-Oxi-αCD NPs (D–F). The morphology of NPs observed by TEM (A,D). The size distribution of NPs determined by DLS (B,E), and the *in vitro* drug release behavior of NPs with or without 0.5 mM H_2_O_2_ (C,F).

**Figure 3.**
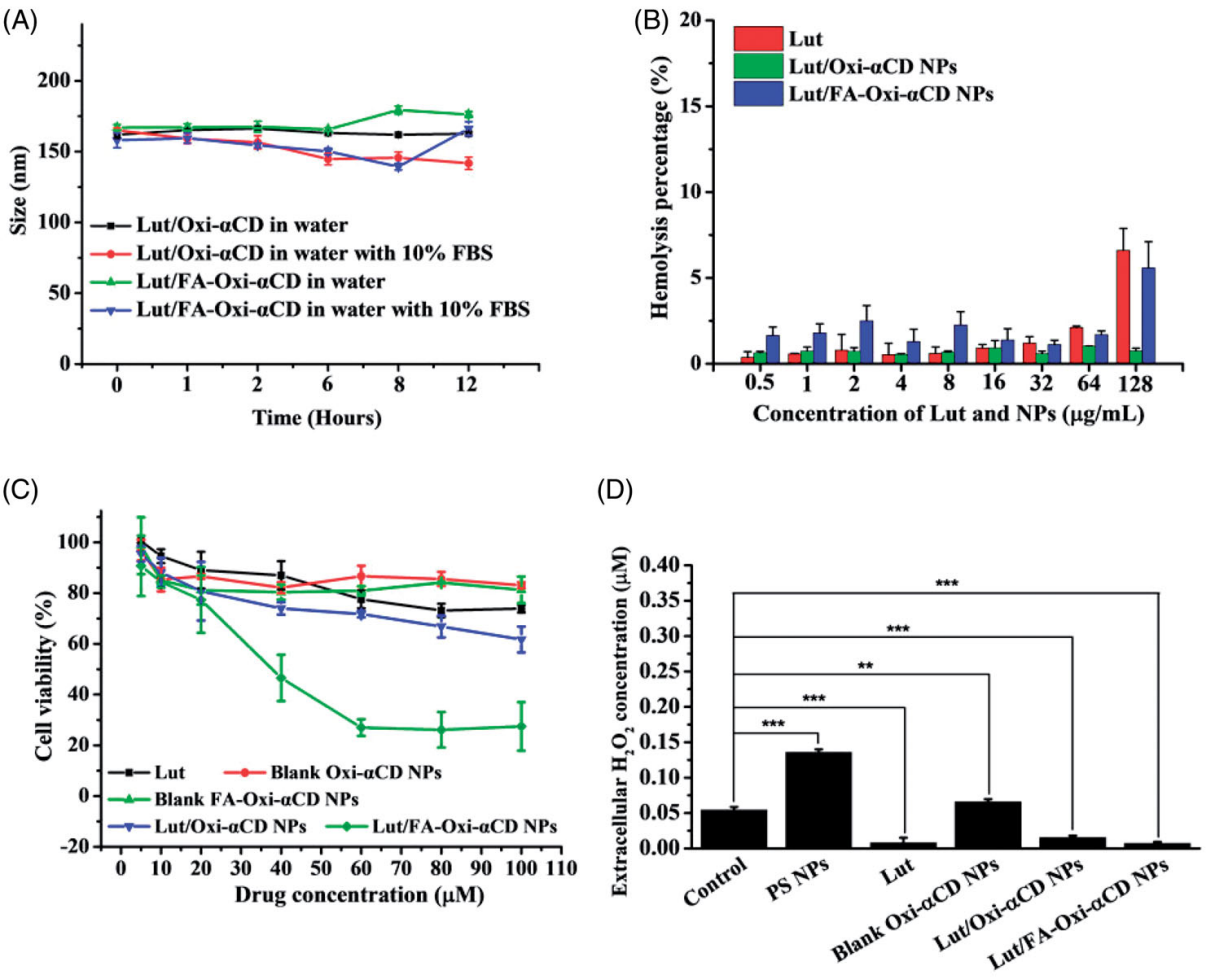
Stability, biocompatibility, *in vitro* anti-tumor activity of Lut-loaded ROS-responsive NPs, and extracellular H_2_O_2_ concentration of 4T1 cells treated with these NPs. (A) The size variability of Lut/Oxi-αCD NPs and Lut/FA-Oxi-αCD NPs in ultrapure water or 10% FBS with different time points; (B) Hemolysis of Lut, Lut/Oxi-αCD NPs and Lut/FA-Oxi-αCD NPs in sheep red blood cells; (C) CCK-8 assays determined the viability of 4T1 cells treated with various NPs for 48 h; and (D) the extracellular H_2_O_2_ concentration of 4T1 cells treated with various nanoformulations. Cells treated with culture medium were used as the control, PS NPs and blank NPs group indicate that cells were treated with polystyrene NPs or Oxi-αCD NPs without Lut-loaded. Each value represents the mean ± *SD* (*n* = 3) (Statistical analysis of Figures 3(C,D) were listed in the Supporting Information, Tables S1, S2). **, statistically different at *p* < .01, ***, statistically different at *p* < .001.

The morphology of Lut/Oxi-αCD NPs and Lut/FA-Oxi-αCD NPs were imaged by TEM. The TEM images indicated that all the NPs were spherical and homogeneous (Figures 2(A,D)). The DLS results also demonstrated that Lut/Oxi-αCD NPs and Lut/FA-Oxi-αCD NPs had uniform size distribution (Figures 2(B,E)). The PDI of all the nanoformulations was lower than 0.2 (Table 1), indicating that these nanoformulations had good dispersity. Alternatively, the stability of these nanoformulations was investigated in ultrapure water with or without 10% FBS. The size of these nanoformulations did not alter obviously within 12 h incubation in the medium (Figure 3(A)). All the nanoformulations had negative zeta potential (Table 1), which could avoid protein absorption on the surface of NPs (Tenzer et al., 2013). To verify whether Oxi-αCD NPs can encapsulate Lut efficiently, the drug loading of Oxi-αCD NPs and FA-Oxi-αCD NPs were tested. Interestingly, the drug loading of Oxi-αCD NPs and FA-Oxi-αCD NPs can reach up to 14.83 ± 3.50 and 16.37 ± 1.47%, respectively (Table 1). Lut/Oxi-αCD NPs and Lut/FA-Oxi-αCD NPs have higher Lut loading compared to other reported Lut nanoformulations (Huang et al., 2017; Qing et al., 2017; Wu et al., 2018), due to the strong affinity of Lut and carrier. Alternatively, the encapsulation efficiencies of Lut in Oxi-αCD NPs and FA-Oxi-αCD NPs were 24.22 ± 2.41 and 33.67 ± 2.43%, respectively (Table 1). To confirm the affinity of Lut and NPs, we analyzed the FT-IR spectrum of Lut, blank NPs, Lut/Oxi-αCD NPs, and Lut/FA-Oxi-αCD NPs. Interestingly, the absorption peak of phenolic hydroxyl of Lut and hydroxyl of blank NPs were both attenuated, which indicated that Lut could form a borate ester bond with Oxi-αCD materials (Figure S1). Simultaneously, the absorption peak of carbonyl and aryl ring were enhanced in Lut/Oxi-αCD NPs and Lut/FA-Oxi-αCD NPs suggested that Lut were encapsulated into the NPs (Figure S1). To simulate the drug release of NPs at physiological conditions, *in vitro* drug release was performed in PBS (0.01 M, pH 7.4) with or without H_2_O_2_. In PBS with 0.5 mM H_2_O_2_, about 80% of Lut was released from Lut/Oxi-αCD NPs within 72 h, while ∼20% of Lut was released in the absence of H_2_O_2_ (Figure 2(C)), suggesting that controlled release of Lut from Oxi-αCD NPs can be performed at diseased sites with high levels of H_2_O_2_. Interestingly, similar drug release behaviors were found between FA-modified nanoformulations and non-targeted nanoformulations (Figures 2(C,F)), implying that FA-modification did not change the drug release profiles of NPs. In conclusion, Lut-loaded ROS-responsive NPs with preferable drug loading and uniform size have been successfully fabricated, and these NPs can smartly release Lut under high levels of ROS.

### 3.2. *In vitro* cytotoxicity, biocompatibility, and anti-tumor activity evaluation

To evaluate blood compatibility of Lut, Lut/Oxi-αCD NPs, and Lut/FA-Oxi-αCD NPs, the hemolysis induced by Lut or NPs treatment was assessed by the sheep blood cells. As shown in Figure 3(B), even the concentration of NPs reached up to 64 μg/mL, the percentage of hemolysis induced by NPs was lower than 3%. Alternatively, the percentage of hemolysis increased slightly when the blood cells were treated with 128 μg/mL of Lut/FA-Oxi-αCD NPs. These results indicated that both Lut/Oxi-αCD NPs and Lut/FA-Oxi-αCD NPs had good blood compatibility. In addition, Lut also exhibited good blood compatibility when its concentration was lower than 64 ug/mL.

As previously described, Lut can inhibit tumor cell proliferation (Imran et al., 2019). Consequently, the cytotoxicity of Lut and its nanoformulations against 4T1 cells were evaluated using the CCK-8 assay. As exhibited in Figure 3(C), blank Oxi-αCD NPs and FA-Oxi-αCD NPs had no *in vitro* anti-tumor activity. However, the viability of 4T1 cells treated with Lut, Lut/Oxi-αCD NPs, or Lut/FA-Oxi-αCD NPs was decreased with the increase of drug concentration (Figure 3(C)). It is worth noting that the *in vitro* anti-tumor activity of Lut and Lut/Oxi-αCD NPs had no significant difference on 4T1 cells (Table S1). On the contrary, the viability of 4T1 cells was decreased significantly when treated with upon 40 µM of Lut/FA-Oxi-αCD NPs (Figure 3(C)), suggesting that FA-modification on the surface of NPs could obviously improve its anti-tumor activity (Table S1). Furthermore, the half-maximal inhibitory concentration (IC_50_) of nanoformulations on 4T1 cells was calculated. The IC_50_ of Lut/Oxi-αCD NPs (about 100 µM) was almost 2.5-fold higher than that of Lut/FA-Oxi-αCD NPs (about 39.9 µM), indicating that Lut/FA-Oxi-αCD NPs had higher cytotoxicity than Lut/Oxi-αCD NPs on 4T1 cells. In summary, cell viability results indicated that Lut-loaded nanoformulations had increased anti-tumor activity than Lut counterpart on 4T1 cells. The reason might be that Lut nanoformulations could be easily internalized by 4T1 cells, and Lut can be smartly released from the NPs under ROS microenvironment.

### 3.3. Intracellular and extracellular H_2_O_2_ of 4T1 cells

As previously reported, Oxi-αCD NPs can influence the intracellular and extracellular H_2_O_2_ concentration of macrophages (Wang et al., 2019; Ni et al., 2020). Herein, the intracellular and extracellular H_2_O_2_ concentration of 4T1 cells mediated by Lut or Oxi-αCD NPs was also investigated. As listed in Figure 3(D), 4T1 cells treated with polystyrene (PS) NPs could significantly increase the extracellular H_2_O_2_ concentration, which was in accordance with the reported results (Bhattacharjee et al., 2014). Interestingly, the extracellular H_2_O_2_ concentration of 4T1 cells was decreased obviously with Lut treatment, due to Lut is a widely used antioxidant that can eliminate ROS (Yan et al., 2016). Alternatively, compared to the control group, the extracellular H_2_O_2_ concentration was increased slightly when cells were treated with blank Oxi-αCD NPs (Figure 3(D)). The reason might be that although Oxi-αCD materials can eliminate ROS, cells treated with NPs can stimulate ROS elevation (Platel et al., 2016). Statistical analysis results indicated that both Lut/Oxi-αCD NPs and Lut/FA-Oxi-αCD NPs could eliminate extracellular ROS dramatically (Table S2). This can be explained that although NPs could stimulate ROS elevation, both Lut and Oxi-αCD NPs could exhaust extracellular ROS. However, both Lut and blank Oxi-αCD NPs could not eliminate intracellular H_2_O_2_ obviously, owing to the concentration of intracellular H_2_O_2_ is far higher than that of extracellular H_2_O_2_, resulting in the H_2_O_2_ concentration variety is not obvious with Lut and Oxi-αCD NPs treatment (Figure S2). Interestingly, intracellular H_2_O_2_ concentration of 4T1 cells was significantly decreased with Lut/Oxi-αCD NPs and Lut/FA-Oxi-αCD NPs treatment (Figure S2), since these NPs were easily internalized by 4T1 cells. Both the internalized Lut and Oxi-αCD NPs could eliminate intracellular H_2_O_2_ dramatically.

### 3.4. Cellular uptake of NPs by 4T1 cells and penetration of NPs in tumor spheroids

**Figure 4.**
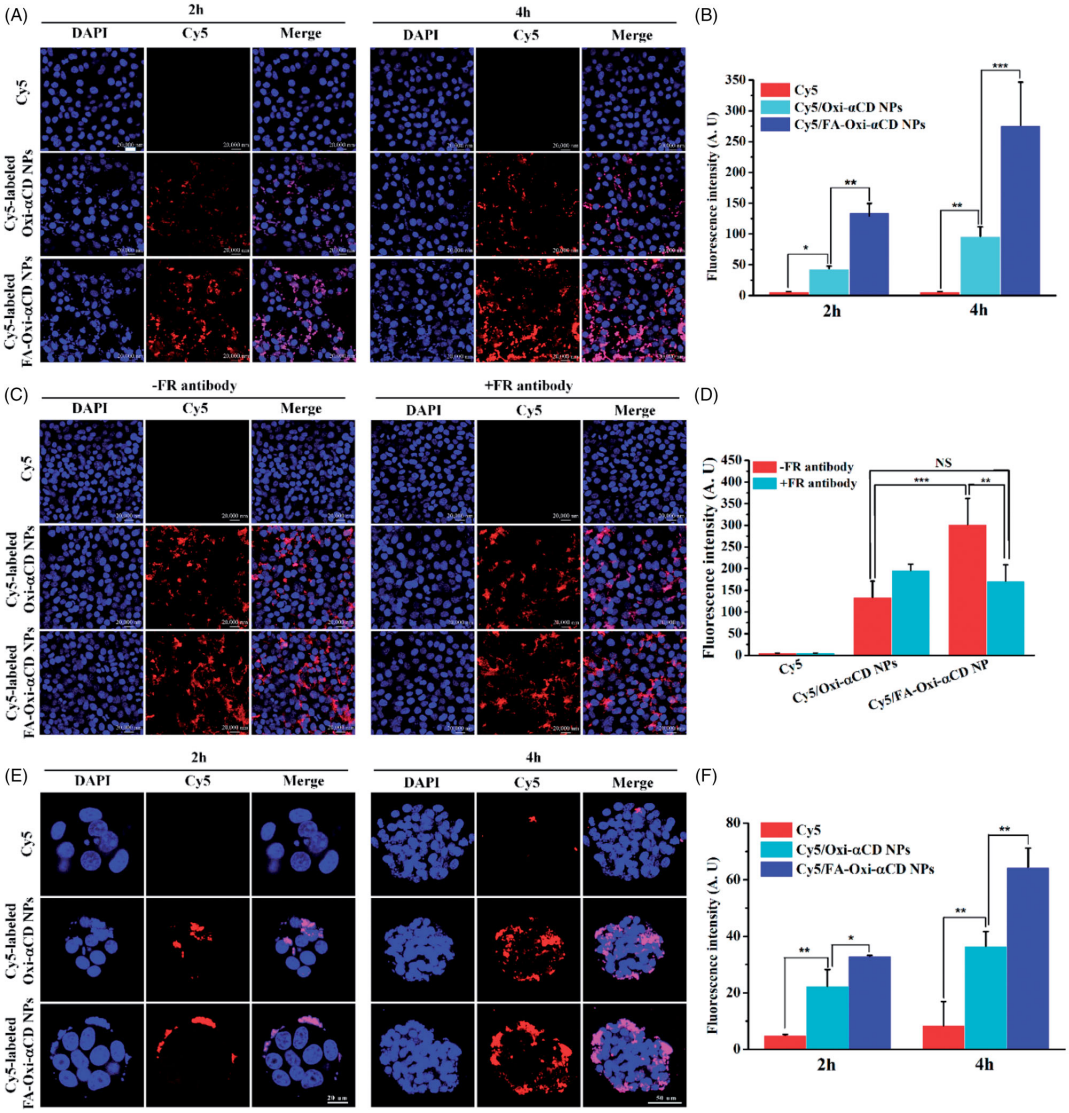
Cellular uptake of Cy5-labeled NPs in 4T1 cells and fluorescence distribution of Cy5-labeled NPs in 4T1 tumor spheroids. (A) Cellular uptake of Cy5-labeled NPs in 4T1 cells after 2 or 4 h treatment. The CLSM images of 4T1 cells incubated with Cy5 (1 μg/mL, red), Cy5/Oxi-αCD NPs or Cy5/FA-Oxi-αCD NPs (containing 1 μg/mL of Cy5) at 37 °C for 2 or 4 h. Cell nuclei were stained with DAPI (blue). Scale bar represents 20 μm. (B) The semi-quantitative analysis of the corresponding Cy5 fluorescence intensity of intracellular NPs (red) is in Figure 4(A). (C) Cellular uptake of Cy5-labeled NPs in 4T1 cells with or without FR antibody treatment. (D) The semi-quantitative analysis of the corresponding Cy5 fluorescence intensity of intracellular NPs (red) in Figure 4(C). (E) Fluorescence distribution of Cy5-labeled NPs (red) in 4T1 tumor spheroids. Tumor spheroid sections were observed at given time points by CLSM. Cell nuclei were stained with DAPI (blue). Scale bar represents 20 μm for 2 h and 50 μm for 4 h. (F) Semi-quantitative analysis of the corresponding Cy5 fluorescence intensity of NPs (red) in tumor spheroid sections. *, statistically different at *p* < .05; **, statistically different at *p* < .01, ***, statistically different at *p* < .001.

As aforementioned, Lut-loaded Oxi-αCD NPs displayed enhanced *in vitro* anti-tumor activity compared to Lut counterpart. To further investigate the *in vitro* anti-tumor activity of Lut-loaded NPs, the cellular uptake of Oxi-αCD NPs by breast cancer 4T1 cells was performed and observed by CLSM. As exhibited in Figure 4(A), compared with free Cy5, Cy5-labeled Oxi-αCD NPs and FA-Oxi-αCD NPs presented strong red fluorescent signals of Cy5 in cells at 2 h, suggesting that Oxi-αCD NPs could be effectively internalized by 4T1 cells. With prolonged incubation time to 4 h, the red fluorescence intensity of Cy5-labeled Oxi-αCD NPs and FA-Oxi-αCD NPs inside 4T1 cells were both significantly increased. Remarkably, Cy5-labeled FA-Oxi-αCD NPs exhibited stronger fluorescence signals than that of non-targeted NPs at 2 and 4 h (Figures 4(A,B)). The reason might be that FRs had been detected on 4T1 cells (Choudhury et al., 2020), which exhibited a strong affinity to FA. To further investigate the affinity of FA-modified NPs with 4T1 cells, 4T1 cells were treated with or without FR antibodies. Interestingly, without FR antibody treatment, FA-modified NPs showed about two times fluorescence intensity than that of non-targeted NPs in 4T1 cells (Figures 4(C,D)). However, there is no significant difference between FA-modified NPs and non-targeted NPs in 4T1 cells when these cells are treated with FR antibodies. These results demonstrated that FA-modification could increase the uptake of NPs by 4T1 cells through FR-mediated endocytosis.

To achieve optimal therapeutic efficacy, NPs need to not only reach tumor sites but also penetrate tumor tissues. Therefore, the penetration abilities of Cy5, Cy5/Oxi-αCD NPs, and Cy5/FA-Oxi-αCD NPs were measured in three dimensional (3 D) tumor spheroid model of breast cancer 4T1 cells. As shown in Figure 4(E), the free Cy5 probe hardly penetrated the core of tumor spheroids and showed weak fluorescence signals around the edge of the spheroids. On contrary, both Cy5-labeled Oxi-αCD NPs and FA-Oxi-αCD NPs exhibited enhanced penetration abilities in 3 D tumor spheroids at 2 and 4 h. Interestingly, fluorescence intensity quantitative analysis indicated that Cy5/FA-Oxi-αCD NPs showed stronger fluorescence signal than Cy5/Oxi-αCD NPs in tumor spheroids (Figure 4(F)), these results were in accordance with cellular uptake results as above described. The excellent penetration abilities of NPs in tumor spheroids may beneficial for targeted drug delivery to the deep of the tumor.

### 3.5. Evaluation of biodistribution *in vivo*

**Figure 5.**
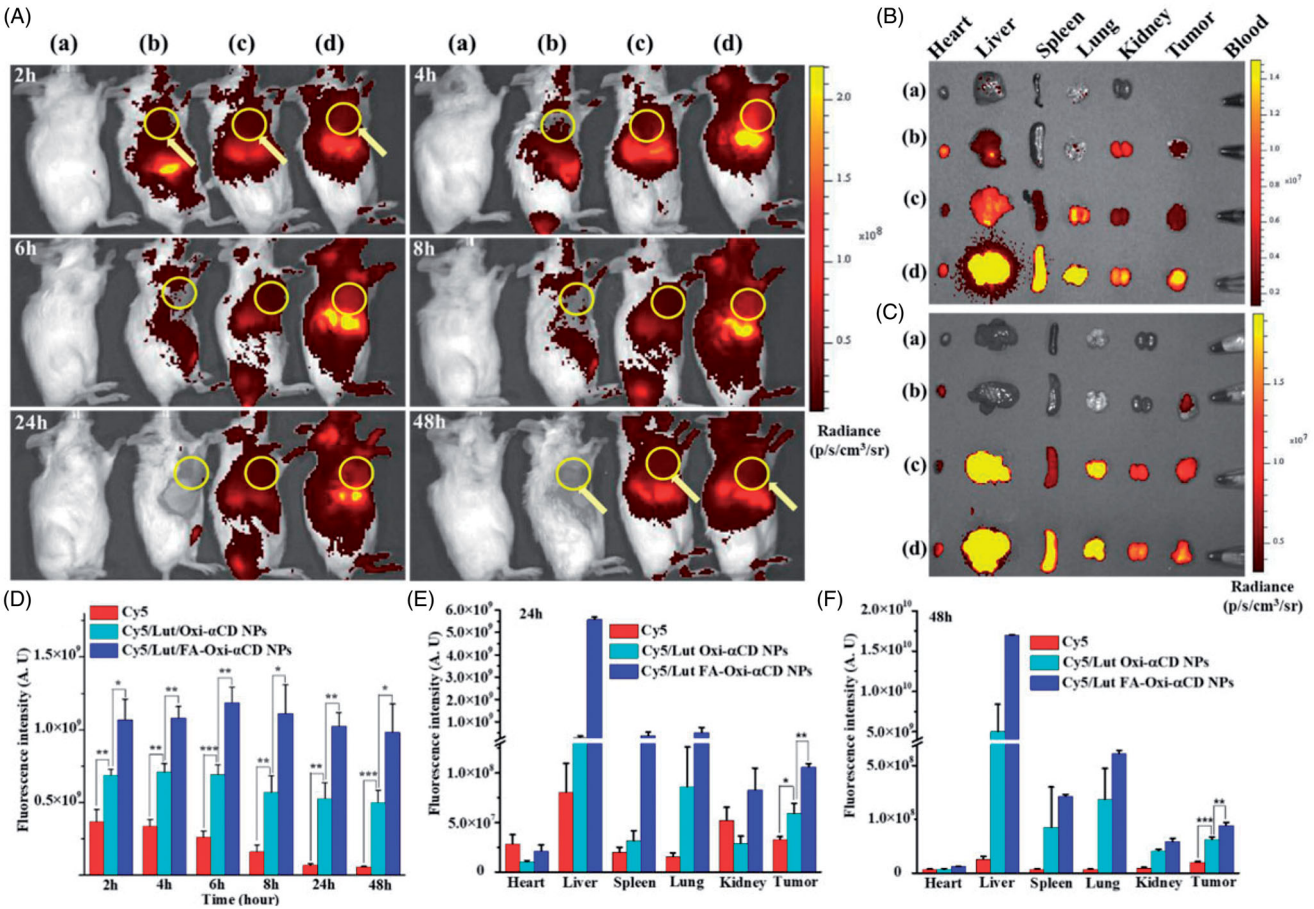
*In vivo* biodistribution of free Cy5, Cy5-labeled Lut/Oxi-αCD NPs and Lut/FA-Oxi-αCD NPs in 4T1 tumor-bearing mice. (A) *In vivo* fluorescence images of free Cy5, Cy5-labeled ROS-responsive NPs in 4T1 tumor-bearing mice at different time points post-intravenous injection, (a) control, (b) free Cy5, (c) Cy5-labeled Lut/Oxi-αCD NPs, and (d) Cy5-labeled Lut/FA-Oxi-αCD NPs (The region in the yellow circle means tumor site). Normal control mice were injected with saline. (B,C) *Ex vivo* fluorescence images of the excised tumors and major tissues at 24 h (B) and 48 h (C) post-injection, (a) control, (b) free Cy5, (c) Cy5-labeled Lut/Oxi-αCD NPs, and (d) Cy5-labeled Lut/FA-Oxi-αCD NPs. (D) Region of Interest (ROI) analysis of fluorescence intensity in tumor tissue was acquired at the interval time points. (E,F) Fluorescence intensities of excised tumor and major tissues, (E) 24 h and (F) 48 h. *, statistically different at *p* < .05; **, statistically different at *p* < .01, ***, statistically different at *p* < .001.

Previous results demonstrated that both Cy5-labeled Oxi-αCD NPs and FA-Oxi-αCD NPs could be efficiently internalized by 4T1 cells and penetrated deeply in 3 D tumor spheroids. Furthermore, the *in vivo* biodistribution and tumor-targeting ability of Cy5-labeled NPs were detected in 4T1 tumor-bearing mouse models using living imaging assay. Compared with the control group, only weak fluorescence signals were detected in the tumor sites of mice treated with free Cy5 after 2 h administration, suggesting that only a few Cy5 dyes accumulated at the tumor sites (Figure 5(A)). On the contrary, both Cy5-labeled Lut/Oxi-αCD NPs and Lut/FA-Oxi-αCD NPs exhibited strong fluorescence signals in tumor regions after 2, 4, 6, 8, 24, and 48 h administration (Figure 5(A)). Importantly, after 48 h post-injection, strong fluorescence intensity was still observed in tumor regions with Cy5-labeled Lut/Oxi-αCD NPs and Lut/FA-Oxi-αCD NPs treatment, indicating that NPs had longer retention time in the body than the free drug. Semi-quantitative analysis indicated that mice that received Cy5-labeled Lut/FA-Oxi-αCD NPs showed the strongest fluorescence intensity in tumor tissues at 2, 4, 6, 8, 24, and 48 h post-injection compared to free Cy5 and non-targeted counterparts (Figure 5(D)). To further analyze the tissues distributions of NPs in the tumor-bearing mice, the isolated primary organs and tumor were subjected to *ex vivo* imaging at 24 h and 48 h post-injection. As shown in Figures 5(B,C), considerable fluorescence signals in liver, spleen, and lung tissues were observed, due to NPs could be trapped by lung, spleen, and liver macrophages (Durymanov et al., 2015). Although these NPs inevitably accumulated in the liver and spleen, the accumulation of NPs in tumor tissues was increased. In accordance with *in vivo* imaging results, FA-modified NPs had stronger fluorescence intensities in tumor tissues compared to that of free Cy5 and non-targeted NPs (Figures 5(E,F)), indicating that FA-modified Oxi-αCD NPs could serve as drug delivery platforms to target deliver Lut to breast tumor.

**Figure 6.**
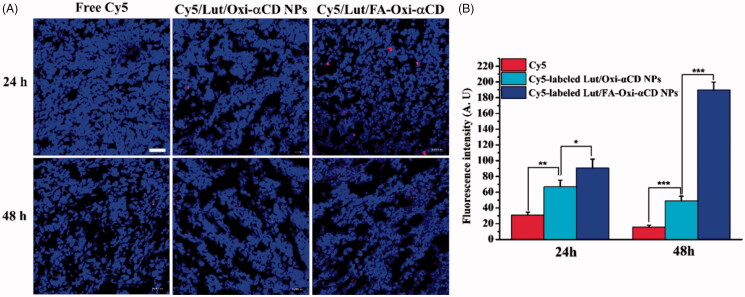
The distribution of free Cy5, Cy5-labeled Lut/Oxi-αCD NPs, and Cy5-labeled Lut/FA-Oxi-αCD NPs in tumor tissues. (A) The CLSM images of tumors treated with Cy5, Cy5-labeled NPs for 24 and 48 h. Red indicates NPs and blue indicates DAPI, respectively. Scale bar represents 50 μm. (B) The semi-quantitative analysis of the corresponding Cy5 fluorescence intensity of NPs (red) in tumor tissue sections. *, statistically different at *p* < .05; **, statistically different at *p* < .01; ***, statistically different at *p* < .001.

Furthermore, the excised tumor tissues were collected for frozen sections and observed by CLSM. The frozen section results also demonstrated that Cy5-labeled Lut/Oxi-αCD NPs and Cy5-labeled Lut/FA-Oxi-αCD NPs could effectively accumulate in the tumor tissues (Figure 6). In accordance with the previous conclusion, compared with Cy5 and Cy5-labeled Lut/Oxi-αCD NPs groups, Cy5-labeled Lut/FA-Oxi-αCD NPs displayed the maximum accumulation in the tumor tissues, suggesting that the nanoplatform could deliver Lut to the interior of tumor tissues.

### 3.6. Initial evaluation of biological safety of Lut and its nanoformulations

Healthy Kunming mice were selected to evaluate the biological safety of Lut and its nanoformulations. As shown in Figure S3, S4 (Supporting Information), the body and organ weight of mice treated by Lut and its nanoformulations had no statistical difference compared to the saline group (Figures S3A,B, S4A,B, Supporting Information). Alternatively, the hematological parameters of blood samples and the concentration of markers related to renal and liver function from mice were not a significant difference between saline and Lut/Oxi-αCD NPs group (Figure S3C–E, Supporting Information). The same results were obtained with Lut/FA-Oxi-αCD NPs treatment except for that creatinine of mice was lower than saline group (Figure S4C–E, Supporting Information). *In vivo* imaging results confirmed that Lut/FA-Oxi-αCD NPs had more accumulation in renal tissues than Lut/Oxi-αCD NPs and free drugs that might affect the creatinine of mice. In conclusion, mice that received 10 mg/kg of Lut or 20 mg/kg Lut-loaded nanoformulations (quantified by Lut) exhibited no obvious adverse effects. Otherwise, examination on hematoxylin-eosin (H&E)-stained sections verified no discernible abnormalities, such as cellular edema, infiltration of inflammatory cells, necrosis, hyperemia, or changes in the morphology of vessels, in the major organs including heart, liver, spleen, lung, and kidney from mice treated with Lut, Lut/Oxi-αCD NPs, and Lut/FA-Oxi-αCD NPs (Figures S5, S6, Supporting Information).

### 3.7. *In vivo* anti-tumor efficacy

**Figure 7.**
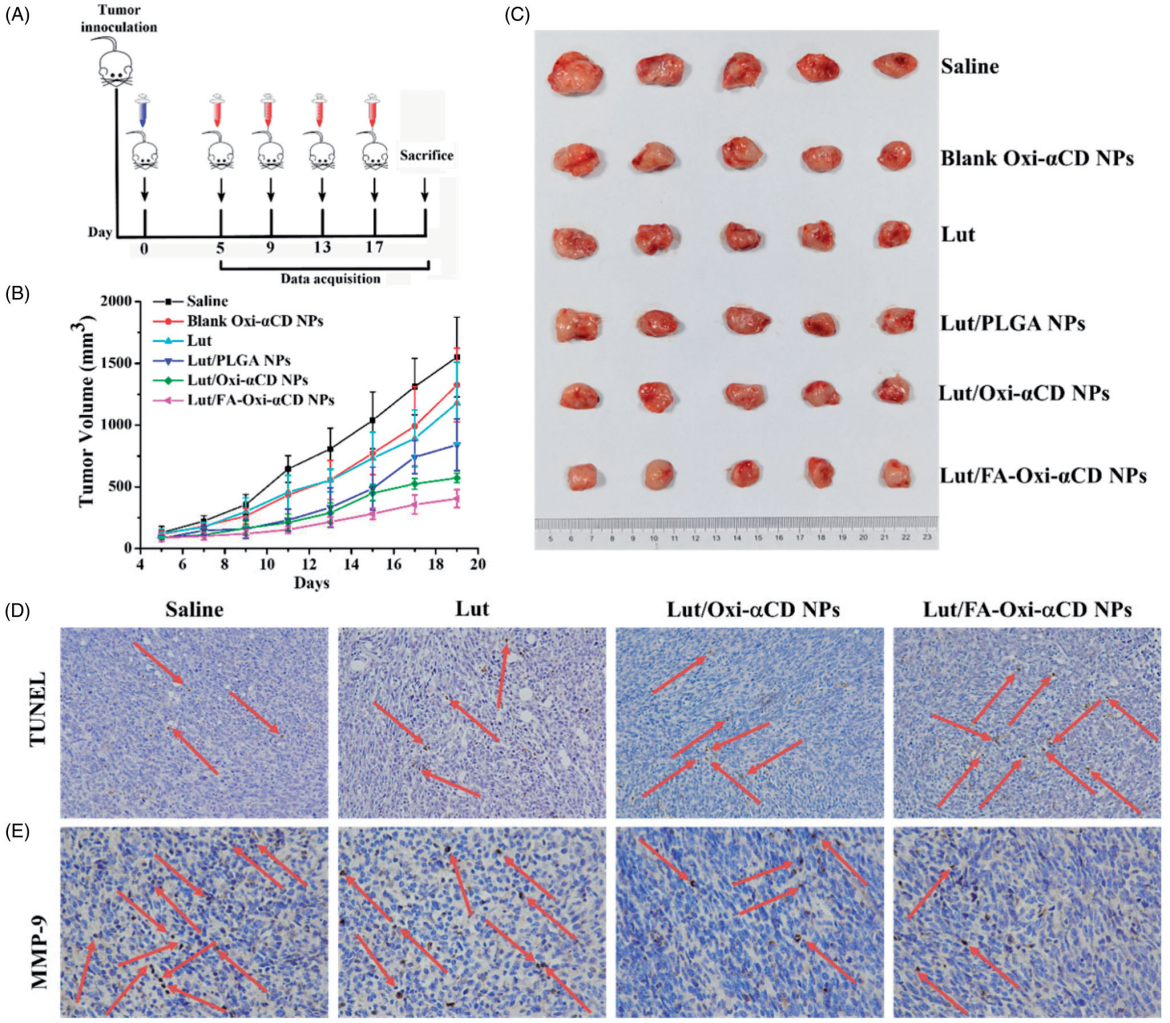
*In vivo* antitumor efficacy evaluation of Lut, blank Oxi-αCD NPs, Lut/PLGA, Lut/Oxi-αCD NPs, and Lut/FA-Oxi-αCD NPs in 4T1 tumor-bearing mice. (A) The administration time after tumor inoculation. Mice received 10 mg/kg of Lut or 10 mg/kg of Lut in nanoformulations every four days. (B) The tumor growth curves after intravenous injection of Lut, blank Oxi-αCD NPs and different Lut nanoformulations, *n* = 5. (C) The excised tumor images with free drug or nanoformulations treatment. (D) TUNEL detection and (E) immunohistochemistry assay for MMP-9 in tumor tissues. Images were obtained with ×200 magnification for TUNEL detection and ×400 magnification for MMP-9 assay. Statistically different was listed in Supporting Information.

Lut/Oxi-αCD NPs, especially Lut/FA-Oxi-αCD NPs exhibited satisfactory *in vitro* anti-tumor activities, and these NPs can accumulate in tumor tissues, which inspires us to investigate these *in vivo* anti-tumor activities. The *in vivo* anti-tumor efficacies of various nanoformulations were assessed using a 4T1 tumor-bearing mouse model. Considering that Lut-loaded NPs can prolong their retention time in the body, tumor-bearing mice received free Lut or Lut nanoformulations every four days (Figure 7(A)). Otherwise, 10 mg/kg of Lut was utilized to evaluate the *in vivo* antitumor activity owing to Lut showed modest *in vivo* antitumor ability. For comparison, Lut-loaded NPs based on non-responsive PLGA material were used as controls. The physicochemical properties of Lut/PLGA NPs were listed in Table 1. All tumor-bearing mice maintained their body weight during the treatment (Figure S7). The tumor volume curves and excised tumor images were shown in Figures 7(B,C). The statistical analysis of *in vivo* antitumor efficacies was listed in Table S3 (Supporting Information). From Table S3, we could find that there is no significant difference when tumor-bearing mice treated with saline and blank Oxi-αCD NPs, indicating that blank Oxi-αCD NPs had no antitumor activities. Lut could inhibit tumor growth compared with the saline group, indicating that Lut had considerable *in vivo* anti-breast cancer activity (Figure 7(B) and Table S3). Lut/Oxi-αCD NPs-treated group showed statistical significance compared to Lut-treated group after 7 days treatment (Table S3), suggesting that the antitumor activity of Lut could be enhanced *via* Oxi-αCD NPs delivery. Interestingly, both Lut/Oxi-αCD NPs and Lut/FA-Oxi-αCD NPs showed stronger anti-tumor activities than Lut/PLGA counterparts (Table S3, Supporting Information), which were in accordance with our previous report (Zhang et al., 2015). Importantly, Lut/FA-Oxi αCD NPs group showed an obvious statistical difference compared with Lut/Oxi-αCD NPs group after 11 days of treatment (Table S3, Supporting Information). These results demonstrated that FA-modification could enhance the antitumor activity of Lut-loaded NPs, which were in accordance with *in vivo* imaging results and *in vitro* antitumor efficacies. The H&E-stained sections of major tissues images indicated that only slight inflammation in the liver was observed with Lut or its nanoformulations treatment (Figure S8, Supporting Information), implying that Lut and its nanoformulations had no significant adverse effects. In addition, the apoptosis of cancer cells after drug treatment was also detected by TUNEL (TdT-mediated dUTP Nick-End Labeling) assay. Both Lut/Oxi-αCD NPs and Lut/FA-Oxi-αCD NPs could obviously induce apoptosis of 4T1 cells compared to saline and Lut group (Figure 7(D)).

In breast cancer, matrix metalloproteinase (MMP) expression is closely related to cancer invasion and metastasis (Dofara et al., 2020). MMP-mediated degradation of the extracellular matrix releases numerous growth factors and signaling molecules that enable tumor expansion. Lut as a potent non-competitive inhibitor of MMP-9, could decrease MMP-9 expression and subsequently reduced the invasive potential of breast cancer cells (Sun et al., 2015). Therefore, we investigated the MMP-9 expression in all therapy groups through the immunostaining method. As shown in Figure 7(E), both Lut/Oxi-αCD NPs and Lut/FA-Oxi-αCD NPs could decrease the levels of MMP-9 obviously compared to the saline and Lut treatment groups, suggesting that Lut-loaded Oxi-αCD NPs had increased anti-tumor activities. These results were consistent with the results of tumor volume measurement (Figure 7(B)). In conclusion, Lut-loaded NPs, especially the Lut/FA-Oxi-αCD NPs could increase the anti-tumor activity of Lut by inducing the apoptosis of the 4T1 cells and reducing the expression of the MMP-9.

## 4. Conclusion

Using ROS-responsive materials Oxi-αCD as a carrier, we successfully fabricated Lut-loaded ROS-responsive NPs. These NPs had spherical structure, uniform size, and satisfying drug loading. In particular, these NPs could smartly release their payloads under pathological ROS levels. The cellular image indicated that both Cy5/Oxi-αCD NPs and Cy5/FA-Oxi-αCD NPs could be internalized by 4T1 cells. The uptake of Cy5/FA-Oxi-αCD NPs was higher than Cy5/Oxi-αCD NPs by 4T1 cells due to FR-mediated endocytosis. *In vivo* imaging results verified that Cy5-labeled Lut/FA-Oxi-αCD NPs had higher accumulation and longer retention at tumor sites compared to free Lut and non-targeted counterparts. Cytotoxicity assays demonstrated that the viability of 4T1 cells was decreased significantly when treated with upon 40 µM of Lut/FA-Oxi-αCD NPs. Importantly, Lut/FA-Oxi-αCD NPs showed about 3-times *in vivo* anti-tumor activities compared to Lut after 15 days of treatment. The reason was that NPs modified by FA could improve the targeting ability of NPs to FR expressed cells which were beneficial to enhance the efficacy of therapeutics. In summary, we successfully developed ROS-responsive nanoplatforms to target delivery Lut to improve its anti-tumor activity. This nano-drug delivery system provides a new strategy to improve the bioactivity of phytopharmaceuticals, such as polyphenol.

## Supplementary Material

Supplemental Material
