# Supplementary material for: Folic acid-modified ROS-responsive nanoparticles encapsulating luteolin for targeted breast cancer treatment
Source: Drug Deliv. 2021 Aug 17;28(1):1695–708. doi: 10.1080/10717544.2021.1963351 (PMC8428179; doi:10.1080/10717544.2021.1963351)
Supplement: Supplemental Material [file IDRD_A_1963351_SM7247.docx]

**Supporting information**

**Materials**

*Synthesis of Oxi-αCD：*Scheme S1 lists the synthesis method of Oxi-αCD [1,2]. Briefly, HPAP (2.00 g, 8.5 mmol) was activated by CDI (2.76 g, 17 mmol) in dry dichloromethane (DCM, 20 mL) to obtain CDI-activated HPAP (2.50 g). CDI-activated HPAP (2.0 g, 6.1 mmol) reacted with α-CD (0.33 g, 0.338 mmol) with DMAP (1.00 g, 8.1 mmol) as a catalyst in DMSO (20 mL) to obtain Oxi-αCD materials (0.735 g). This materials was confirmed by ^1^H NMR spectra.

Scheme S1, synthesis of Oxi-αCD

[1] Wang, Y.; Yuan, Q.; Feng, W.; Pu, W. D.; Ding, J.; Zhang, H. J.; Li, X. Y.; Yang, B.; Dai, Q.; Cheng, L.; Wang, J. Y.; Sun, F. J.; Zhang, D. L., Targeted delivery of antibiotics to the infected pulmonary tissues using ROS-responsive nanoparticles. *J Nanobiotechnol* **2019,** *17* (1), 103.

[2] Ni, R. R.; Song, G. J.; Fu, X. H.; Song, R. F.; Li, L. L.; Pu, W. D.; Gao, J. N.; Hu, J.; Liu, Q.; He, F. T.; Zhang, D. L.; Huang, G., Reactive oxygen species-responsive dexamethasone-loaded nanoparticles for targeted treatment of rheumatoid arthritis via suppressing the iRhom2/TNF-alpha/BAFF signaling pathway. *Biomaterials* **2020,** *232*, 119730.

*Synthesis of Cy5-labeled Oxi-αCD:* The synthesis method of Cy5-labeled Oxi-αCD was exhibited in scheme S2 [1,2]. Cy5 free acid (5.0 mg, 0.00963 mmol) was dissolved in 5.0 mL of DMF and activated by DMAP (2.0 mg, 0.0164 mmol) and EDC. HCl (7.4 mg, 0.0385 mmol). Then 50.0 mg of α-CD (0.0514 mmol) was added into above solution and reacted at 25 °C for 2 days. After removal of the organic solvent, the residue was washed with acetone to obtain Cy5-conjugated α-CD. Using DMAP (150.0 mg 1.216 mmol) as a catalyst, Cy5-conjugated α-CD (50.0 mg) was reacted with CDI-activated HPAP (300.0 mg, 0.915 mmol) to obtain Cy5-labeled Oxi-αCD.

Scheme S2, synthesis of Cy5-labeled Oxi-αCD

[1] Wang, Y.; Yuan, Q.; Feng, W.; Pu, W. D.; Ding, J.; Zhang, H. J.; Li, X. Y.; Yang, B.; Dai, Q.; Cheng, L.; Wang, J. Y.; Sun, F. J.; Zhang, D. L., Targeted delivery of antibiotics to the infected pulmonary tissues using ROS-responsive nanoparticles. *J Nanobiotechnol* **2019,** *17* (1), 103.

[2] Ni, R. R.; Song, G. J.; Fu, X. H.; Song, R. F.; Li, L. L.; Pu, W. D.; Gao, J. N.; Hu, J.; Liu, Q.; He, F. T.; Zhang, D. L.; Huang, G., Reactive oxygen species-responsive dexamethasone-loaded nanoparticles for targeted treatment of rheumatoid arthritis via suppressing the iRhom2/TNF-alpha/BAFF signaling pathway. *Biomaterials* **2020,** *232*, 119730.

**Fabrication of NPs**

*Cy5-labeled Oxi-αCD:* Lecithin (4.0 mg) and DSPE-PEG_2000_ (6.0 mg) were dispersed in anhydrous ethanol (400 µL) and deionized water (10.0 mL), and then heated to 65 °C for 30 min under gentle stirring (100 rpm). In parallel, Cy5 labeled Oxi-αCD (5.0 mg) and Oxi-αCD (45.0 mg) were dissolved in methanol (1.0 mL) and dimethyl sulfoxide (DMSO) (1.0 mL). The carrier-containing solution was dropped into the above preheated lipid dispersion solution (1.0 mL/min) followed by vortexing for 3 min (800 rpm). After self-assembly with stir slowly for 2 h at room temperature, Cy5-labeled Oxi-αCD NPs were collected by centrifugation at 15000 rpm for 10 min. The harvested NPs were washed with 5 % F127 (10.0 mL) and resuspended in 0.2 mL ultrapure water. All the experiment procedures were conducted under the dark conditions.

An emulsion solvent evaporation method was employed to prepare Lut/PLGA NPs. Briefly, 50 mg of PLGA was dissolved in 0.6 mL dichloromethane (DCM). 5 mg of Lut was dissolved in 0.15 mL of DMSO and then dropped slowly into the PLGA solution. The organic solution was emulsified into 7 mL of PVA aqueous solution (1.0 wt. % in PBS at pH 7.4) via probe sonication to form nanoemulsion. Then the formed emulsion was poured into 20 mL of PVA aqueous solution (0.3 wt.% in PBS at pH 7.4) and magnetically stirred at room temperature for 2 h to remove DCM. The solidified NPs were harvested by centrifugation at 15,000 rpm for 10 min, and washed with deionized water 3 times [1]. The Lut content loaded in PLGA NPs was determined by HPLC.

[1] Dinglin Zhang, Yanling Wei, Kai Chen, Hao Gong, Songling Han, Jiawei Guo, Xiaohui Li, and Jianxiang Zhang. Engineering of Biocompatible pH-Responsive Nanovehicles from Acetalated Cyclodextrins as Effective Delivery Systems for Tumor Therapy. Journal of Biomedical Nanotechnology, 2015, 11: 923-941.

**Results**

**Table S1.** Statistical analysis of *in vitro* antitumor data in Figure 3C.

| Concentration (μM) | Groups | | | |
| --- | --- | --- | --- | --- |
|  | Lut | Blank NPs | Lut/Oxi-αCD NPs | Lut/FA-Oxi αCD NPs |
| 5 | - | - | - | - |
| 10 | - | - | - | - |
| 20 | - | - | - | - |
| 40 | - | - | - | *** p < 0.001  ^+++^ p < 0.001  ^###^ p < 0.001 |
| 60 | - | - | ^+^ p < 0.05 | ***p<0.001  ^+++^ p < 0.001  ^###^ p < 0.001 |
| 80 | - | - | ^++^ p < 0.01 | ***p<0.001  ^+++^ p < 0.001  ^###^ p < 0.001 |
| 100 | - | - | ^+++^ p < 0.001 | *** p < 0.001  ^+++^ p < 0.001  ^###^  p < 0.001 |

* p < 0.05, ** p < 0.01, and *** p < 0.001 versus the Lut group;

^+^ p < 0.05, ^++^ p < 0.01, and ^+++^ p < 0.001 versus the Blank NPs group;

^#^ p < 0.05, ^##^ p < 0.01, and ^###^ p < 0.001 versus the Lut/Oxi-αCD NPs group;

**Table S2.** Statistical analysis of H_2_O_2_ concentration in Figure 3D.

|  | Control | PS NPs | Lut | Blank NPs | Lut/Oxi-αCD NPs | Lut/FA-Oxi αCD NPs |
| --- | --- | --- | --- | --- | --- | --- |
| Control | - | *** | *** | ** | *** | *** |
| PS NPs | *** | - | *** | *** | *** | *** |
| Lut | *** | *** | - | *** | ns | ns |
| Blank NPs | ** | *** | *** | - | *** | *** |
| Lut/Oxi-αCD NPs | *** | *** | ns | *** | - | * |
| Lut/FA-Oxi αCD NPs | *** | *** | ns | *** | * | - |

* p < 0.05, ** p < 0.01, and *** p < 0.001;

**Table S3.** Statistical analysis of *in vivo* antitumor data in Figure 7B.

| Time (days) | Groups | | | | | |
| --- | --- | --- | --- | --- | --- | --- |
|  | Saline | Blank Oxi-αCD NPs | Lut | Lut/PLGA | Lut/Oxi-αCD NPs | Lut/FA-Oxi-αCD NPs |
| 1 | - | - | - | - | - | - |
| 3 | - | - | - | - | - | - |
| 5 | - | - | - | - | - | - |
| 7 | - | - | - | *** *p*<0.001 | *** *p*<0.001  ^#^ *p* < 0.05 | *** *p*<0.001  ^+^ *p* < 0.05  ^##^ *p* < 0.01 |
| 9 | - | - | - | *** *p*<0.001 | *** *p*<0.001  ^+^ *p* < 0.05  ^#^ *p* < 0.05 | *** *p*<0.001  ^++^ *p* < 0.01  ^###^ *p* < 0.001 |
| 11 | - | - | - | *** *p*<0.001 | *** *p*<0.001  ^++^ *p* < 0.01  ^##^ *p* < 0.01 | *** *p*<0.001  ^+++^ *p* < 0.001  ^###^ *p* < 0.001  ^$^ *p* < 0.05  ^&&&^ *p* < 0.001 |
| 13 | - | - | ***p*<0.01 | *** *p*<0.001 | *** *p*<0.001  ^+++^ *p* < 0.001  ^###^ *p* < 0.001  ^$$^ *p* < 0.01 | *** *p*<0.001  ^+++^ *p* < 0.001  ^###^ *p* < 0.001  ^$$$^ *p* < 0.001  ^&&&^ *p* < 0.001 |
| 15 | - | - | * *p* < 0.05 | *** *p*<0.001  ^+++^ *p* < 0.001  ^##^ *p* < 0.01 | *** *p*<0.001  ^+++^ *p* < 0.001  ^###^ *p* < 0.001  ^$$$^ *p* < 0.001 | *** *p*<0.001  ^+++^ *p* < 0.001  ^###^ *p* < 0.001  ^$$$^ *p* < 0.001  ^&&&^ *p* < 0.001 |

* *p* < 0.05, ** *p* < 0.01, and *** *p* < 0.001 *versus* the saline group;

^+^ *p* < 0.05, ^++^ *p* < 0.01, and ^+++^ *p* < 0.001 *versus* the blank group;

^#^ *p* < 0.05, ^##^ *p* < 0.01, and ^###^ *p* < 0.001 *versus* the lut group;

^$^ *p* < 0.05, ^$$^ *p* < 0.01, and ^$$$^ *p* < 0.001 *versus* the Lut/PLGA group;

^&^ *p* < 0.05, ^&&^ *p* < 0.01, and ^&&&^ *p* < 0.001 *versus* the Lut/Oxi-αCD NPs group;

Figure S1. IR spectrum of Lut, blank Oxi-αCD NPs, Lut/Oxi-αCD NPs and Lut/FA-Oxi-αCD NPs.

Figure S2. Intracellular H_2_O_2_ concentration of 4T1 cells treated with Lut and various nanoformulations. *, statistically different at P < 0.05; **, statistically different at P < 0.01, ***, statistically different at P < 0.001.


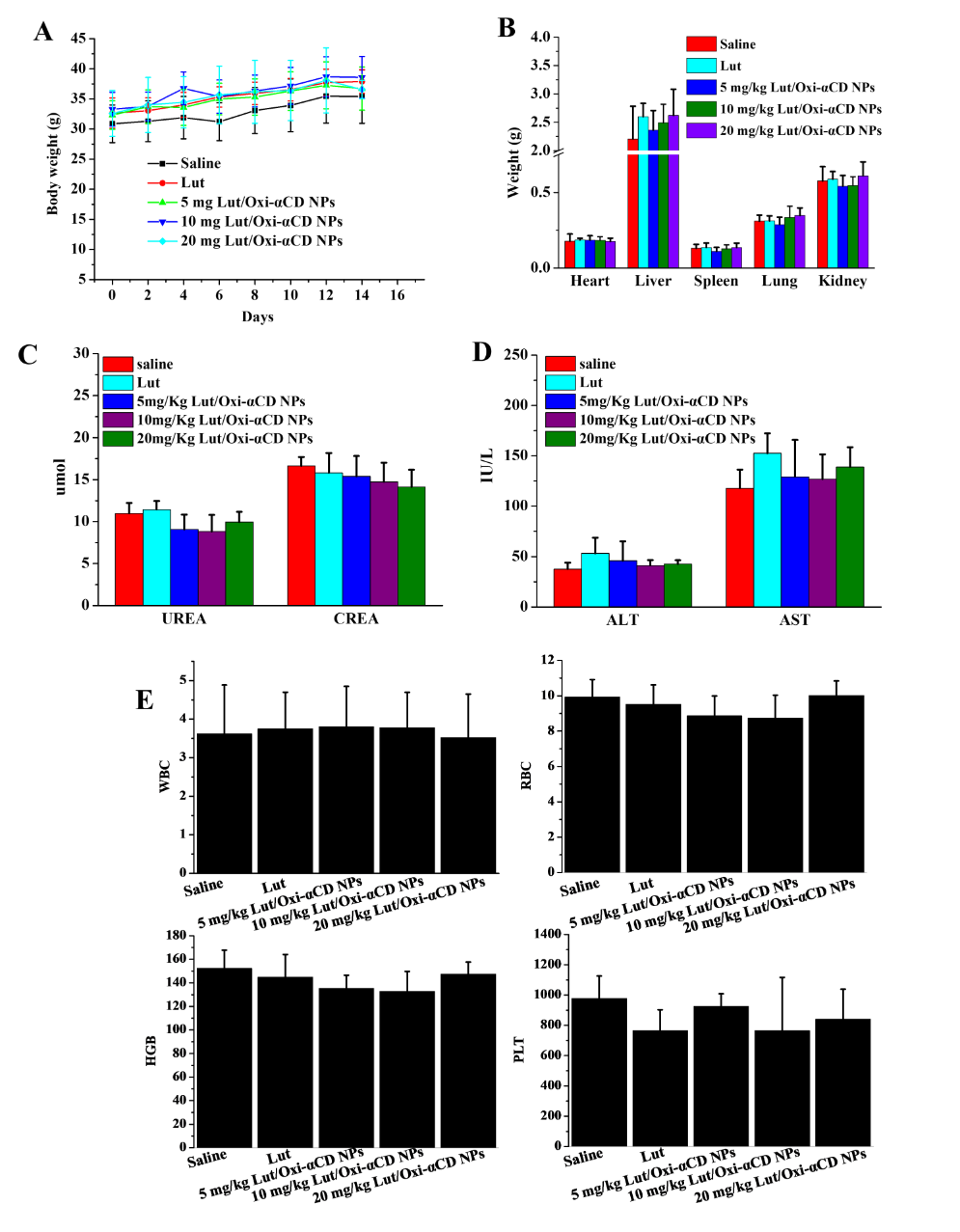


Figure S3, Initial evaluation of biological safety of Lut/Oxi-αCD NPs after *i.v.* administration at various doses. (A) Body weight, (B) weight of organ, (C) The concentration of markers related to renal function. CREA, creatinine; and UREA, urea. (D) The levels of two biochemical markers relevant to liver functions. ALT, alanine aminotransferase; and AST, aspartate aminotransferase. (E) Hematological parameters of blood samples from mice subjected to various treatments. WBC, white blood cell; HGB, hemoglobin; RBC, red blood cell; and PLT, platelet. Data are mean ± SD (n = 6). All data has no statistical difference.


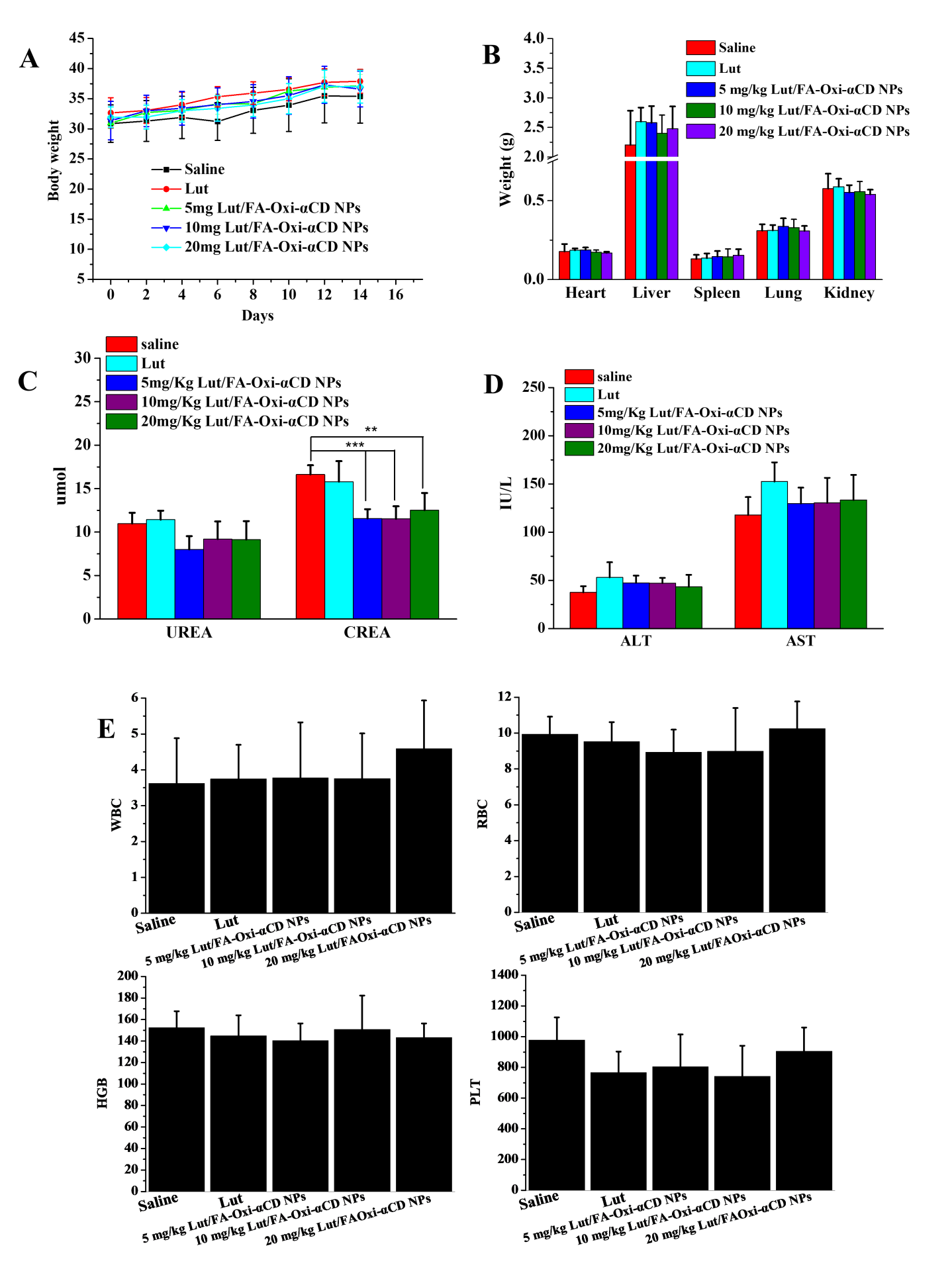


Figure S4, Initial evaluation of biological safety of Lut/FA-Oxi-αCD NPs after *i.v.* administration at various doses. (A) Body weight, (B) weight of organ, (C) The concentration of markers related to renal function. CREA, creatinine; and UREA, urea. (D) The levels of two biochemical markers relevant to liver functions. ALT, alanine aminotransferase; and AST, aspartate aminotransferase. (E) Hematological parameters of blood samples from mice subjected to various treatments. WBC, white blood cell; HGB, hemoglobin; RBC, red blood cell; and PLT, platelet. Data are mean ± SD (n = 6). * p < 0.05, ** p < 0.01, and *** p < 0.001 versus the saline group.


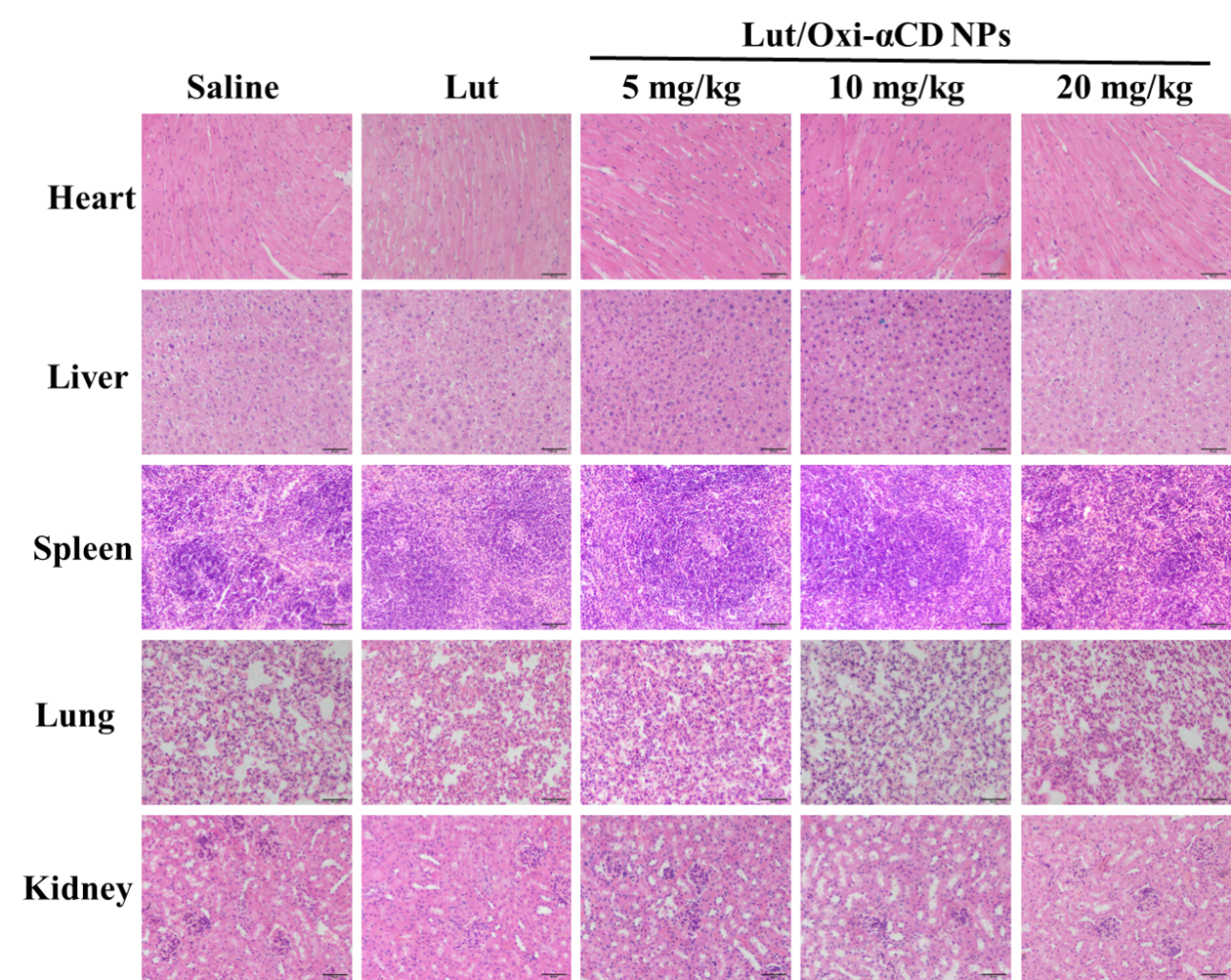


Figure S5. Histological examination of the major organs from healthy mice with saline, Lut and Lut/Oxi-αCD NPs treatment by H&E staining. Images were obtained with ×100 magnification.


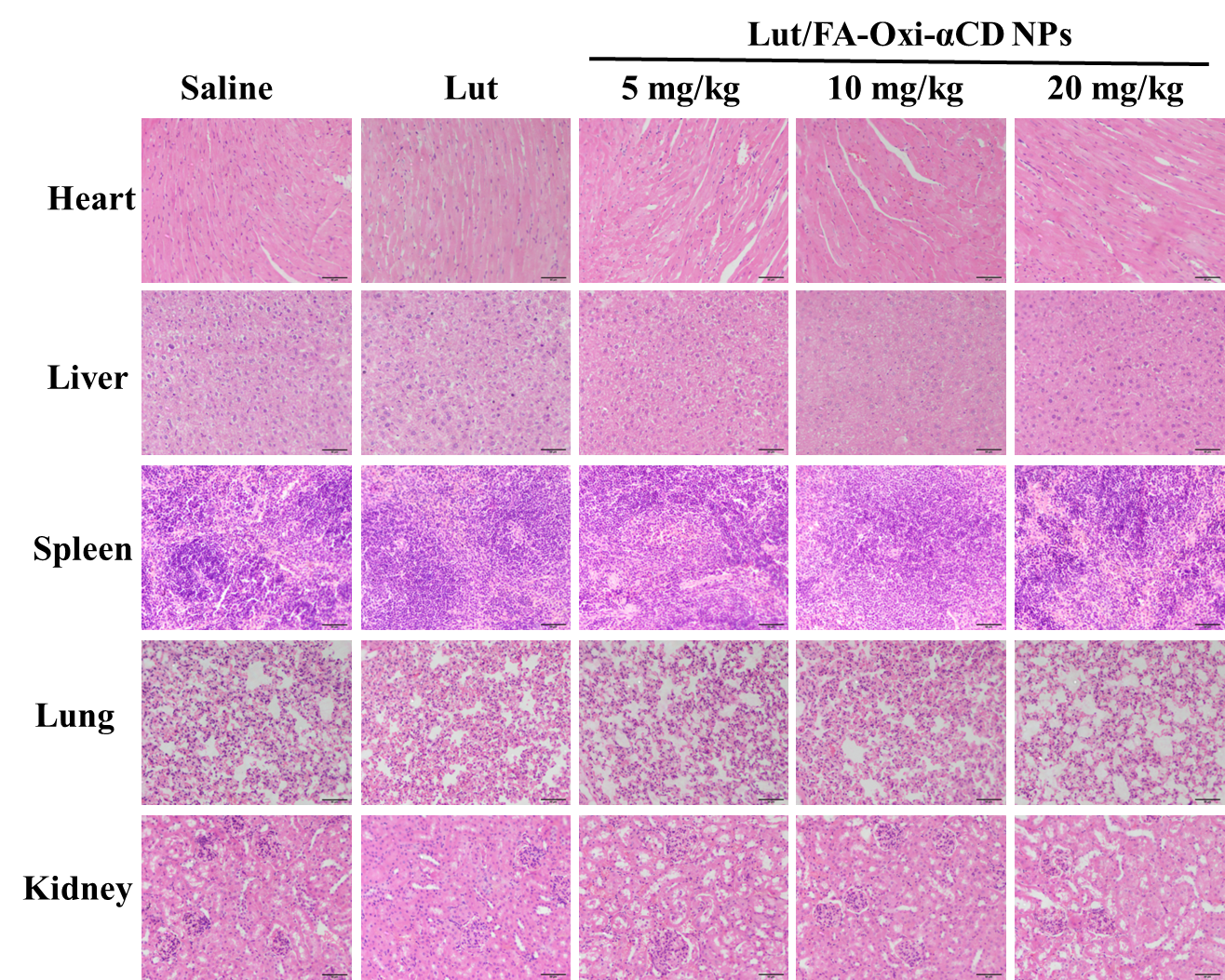


Figure S6. Histological examination of the major organs from healthy mice with saline, Lut and Lut/FA-Oxi-αCD NPs treatment by H&E staining. Images were obtained with ×100 magnification.

Figure S7. Body weight of tumor-bearing mice treated with Lut and its various nanoformulations.


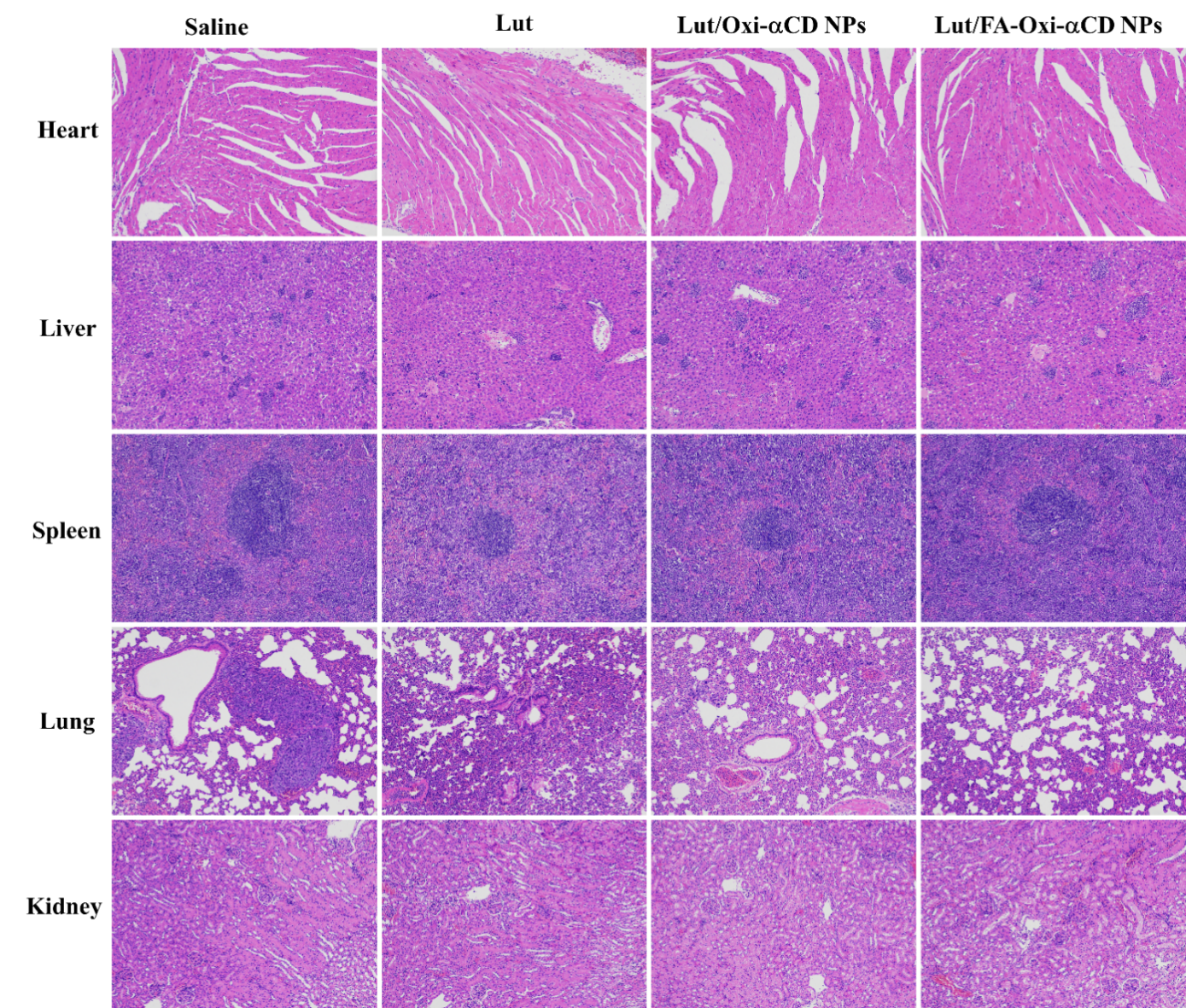


Figure S8. Histological examination of the major organs from tumor-bearing mice with saline, Lut, Lut/Oxi-αCD NPs and Lut/FA-Oxi-αCD NPs treatment by H&E staining. Images were obtained with ×100 magnification.
